# Risk Factors for the Development of Barrett's Esophagus and Esophageal Adenocarcinoma: A Systematic Review and Meta‐Analysis

**DOI:** 10.1002/cnr2.70168

**Published:** 2025-03-04

**Authors:** Kais Antonios, Daniel Aintabi, Patricia McNally, Elliot Berinstein, Priyata Dutta, Nicholas Sampson, Sichao Wang, Claudia Villarreal Carrillo, Brahm Singh, Marjan Haider, Richard A. Shellenberger

**Affiliations:** ^1^ Trinity Health Ann Arbor Hospital Ann Arbor Michigan USA; ^2^ Internal Medicine Trinity Health Ann Arbor Hospital Ypsilanti Michigan USA; ^3^ Michigan State University East Lansing Michigan USA

**Keywords:** Barrett's esophagus, cancer epidemiology, cancer screening, early detection, esophageal adenocarcinoma, gastroesophageal reflux

## Abstract

**Background:**

Barrett's esophagus (BE) is the most widely established precursor to esophageal adenocarcinoma (EAC). Despite current screening guidelines, more than 90% of EAC patients lack a previous diagnosis of BE. We performed a systematic review and meta‐analysis to identify the most important risk factors for the development of BE or EAC.

**Recent Findings:**

PubMed.gov, Ovid Medline, Embase, and Cochrane Library were searched through March 15, 2024. Studies comparing characteristics of patients with endoscopically diagnosed BE or EAC to control groups satisfied our inclusion criteria. Dual extraction provided data for random‐effects meta‐analyses. Sufficient data were extracted from 54 included studies to perform our meta‐analyses. There were five risk factors with significant associations for the development of BE: symptoms of gastroesophageal reflux at least once weekly (OR, 3.56; 95% confidence interval [CI], 2.03–6.25; *p* = 0.004) tobacco smoking (OR, 1.41; 95% CI, 1.30–1.51; *p* < 0.001); alcohol use (OR, 1.37; 95% CI, 1.10–1.71; *p* = 0.008); male gender (OR, 1.36; 95% CI, 1.19–1.57; *p* < 0.001); and obesity (BMI > 30 kg/m^2^) (OR, 1.23; 95% CI, 1.09–1.39; *p* = 0.003). Tobacco smoking was significantly associated with the diagnosis of EAC (OR, 2.15; 95% CI, 1.85–2.43; *p* < 0.001).

**Conclusion:**

Five risk factors showed significant associations with the development of BE and one with the development of EAC, with over a three‐fold increase in BE for patients with gastroesophageal reflux more than once weekly. These data could prove useful in developing diagnostic paradigms with higher emphasis on patients experiencing more frequent acid reflux.

## 1 Introduction

Esophageal adenocarcinoma (EAC) has a rising incidence rate and mortality rate in North America, much of Western and Northern Europe, and Oceania over the past three decades, and has become the most prevalent form of esophageal cancer in the developed world [1, 2]. In the United States, the incidence rate of EAC has risen seven‐fold from 1975 to 2017 [3, 4]. Barrett's esophagus is well recognized as the most prevalent precursor to the development of EAC. Despite surveillance programs for patients with a diagnosis of Barrett's esophagus (BE) and advances in cancer treatment, improvements in the prognosis of EAC have not been demonstrated worldwide, with a five‐year survival of only 25% [5]. Since the presence of clinical signs of EAC usually becomes apparent in late‐stage disease, the opportunity of identifying early‐stage disease relies on identifying individual patients who are at higher risk for the development of this cancer [6]. Although screening guidelines for the detection of BE exist in the United States and Europe, they have not been consistently implemented to improve early detection of EAC [7]. In fact, in a US cohort of 8564 EAC patients from the Veterans Affairs Central Cancer registry diagnosed between 2002 and 2017, only 4.9% had a prior diagnosis of BE [8].

Established guidelines for BE diagnosis and screening include three in the United States [9, 10, 11], one in the United Kingdom [12], and one in Europe [13]. Each of these guidelines gives recommendations on endoscopic evaluation for BE in at‐risk patients based on limited or low‐quality evidence. All five of the guidelines use gastroesophageal reflux disease (GERD), age > 50 years, male gender, and obesity as risk factors for the development of BE, and a diagnostic evaluation is recommended only in patients with multiple risk factors. Since BE is typically diagnosed in symptomatic patients who receive an upper endoscopy and biopsy, it is difficult to define the prevalence of this condition in the general population, which makes it difficult to establish widespread screening practices. From a large systematic review and meta‐analysis, the global prevalence of BE is less than 1%; however, patients with GERD have been shown to have a prevalence of BE as high as 7.8% [14]. The dilemma is that 40% of EAC patients in the United States do not have a history of reflux, whereas 52% have a history of GERD but did not undergo an endoscopic evaluation [15]. In fact, only 7% of EAC patients in the United States have a prior diagnosis or are part of a surveillance program for BE [15]. Based on these data, it is apparent that an evidence‐based strategy based on risk stratification could be useful for enhancing the diagnosis of BE.

The purpose of our systematic review and meta‐analysis was to strengthen the existing database pertaining to risk factors and clinical predictors for the development of BE and EAC. Most importantly, these risk factor data may become an addition to current clinical practice guidelines and assist in maximizing the diagnosis of patients with BE. The aim of our study is to present a new database that could improve the selection of patients who should have diagnostic endoscopies due to their high risk for the development of BE.

## 2 Methods

The following methodology has been followed with strict adherence to ensure the accuracy of our study and is reported in accordance with the Preferred Reporting Items for Systematic Reviews and Meta‐Analysis (PRISMA) Guidelines [16]. Our study protocol is registered with PROSPERO (ID: CRD42023428440) (Supporting Information S1).

### 2.1 Data Sources and Searches

Our database was populated through common indexing practices to capture potentially relevant publications. PubMed.gov, Ovid Medline, Embase, and Cochrane Library were searched from their inception dates until March 15, 2024, without language restrictions. The keywords, medical subject heading terms, and search strategies we employed are available elsewhere (Supporting Information S1). Appropriate studies were added through reference review from selected articles.

### 2.2 Study Selection

Two independent investigators (K.A. and R.A.S.) compiled a reference list that was uploaded into file management software. Duplicates were removed, and titles and abstracts were screened for eligibility criteria. Studies potentially meeting inclusion criteria were saved for full‐text review after a consensus agreement by both investigators.

Randomized‐control trials, prospective cohort observational studies, and case‐control observational studies were preferred due to a lower risk of bias. To maximize the identification of risk factors, we included studies that described any patient characteristics identified in study subjects with either BE or EAC and compared them with either healthy or endoscopic control patients. We excluded studies without control group comparisons to mitigate the risk of bias. Patient characteristics were those widely identifiable in standard primary care practices across a global spectrum of health care systems, as we excluded any studies that examined the relationship of BE or EAC with genetic testing or laboratory testing not routinely available. Abstract‐only papers or posters were excluded due to the lack of a thorough description of study design and methods of data acquisition.

### 2.3 Data Extraction and Quality Assessment

All included studies had data extracted through consensus agreement by two investigators (R.A.S. and D.A.). The same two investigators independently assessed the quality and risk of bias of all included studies using six domains from the Newcastle‐Ottawa Quality Assessment Tool for Observational, Cohort, and Cross‐Sectional Studies available from the National Institute of Health [17]. The certainty of evidence was evaluated for each study by using the Grading of Recommendations Assessment, Development, and Evaluation (GRADE) Working Group framework assessment [18].

### 2.4 Data Synthesis

Whenever data from studies were sufficient and of similar design, participants, and outcomes, we performed meta‐analyses to yield quantitative estimates that required pooling to increase precision. We anticipated our data from case‐control and cohort studies would have a high degree of variability in patient populations and demographics and planned to employ a random effects model for all meta‐analyses. Odds ratios (OR) were calculated with the Hartung‐Knapp adjustment and inverse variance weighting in an attempt to determine a single effect size for each variable that had a sufficient number of studies [19]. Heterogeneity was statistically evaluated by the Paule‐Mandel estimate for tau squared with the Q‐Profile method for confidence intervals (CI). The continuity correction of 0.5 was employed in studies with zero cell frequencies. Publication bias was assessed for each risk factor in our meta‐analysis, with sensitivity analysis performed using the leave‐one‐out method when outliers were identified on funnel plots (Figure S1). Outliers were identified when the CI failed to overlap with the CI of the pooled effect. All statistical analysis was carried out using the meta package (version 6.5‐0) in R Statistical Software (version 4.3) [20].

### 2.5 Ethics Statement

The institutional review board at Trinity Health Ann Arbor Hospital, Ann Arbor, MI, deemed our study to be exempt from their ethical review process.

## 3 Results

**FIGURE 1:**
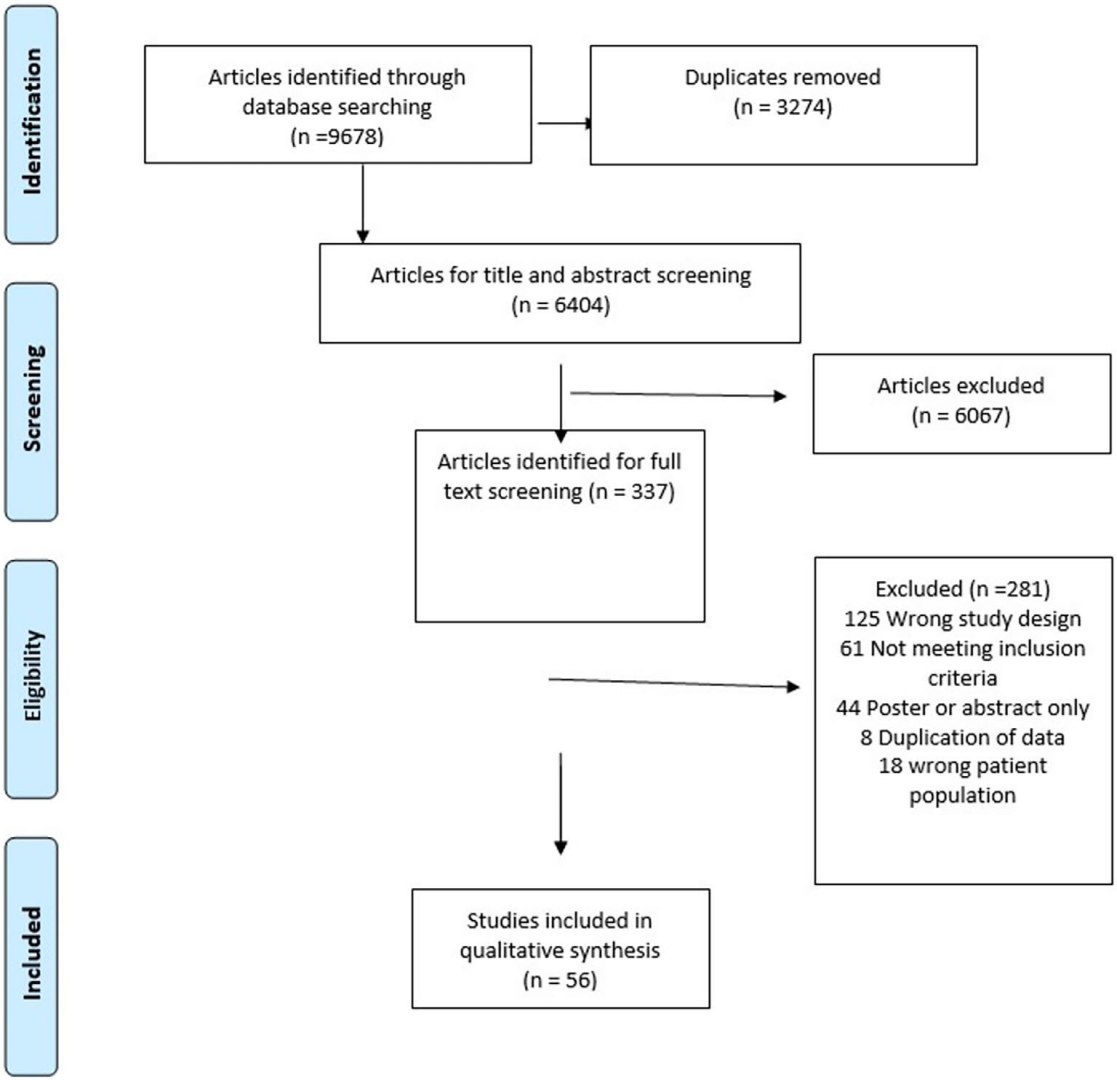
PRISMA flow diagram.

The literature search identified 9768 articles. After deleting duplicates, we compiled 6404 studies for title and abstract screening for eligibility in our systematic review (Figure 1). Following PRISMA guidelines for dual extraction, two investigators (K.A. and R.A.S.) identified 337 studies for full‐text review, with 56 of them meeting our inclusion criteria to have data extracted for statistical analysis. Data was sufficient to perform meta‐analyses on 54 of these studies whose characteristics included: first author and date of study; study setting and location; study design; risk factors assessed for the development of BE or EAC; number of case and control patients; mean age of the case patients; and the type of control group (Table 1). We had sufficient data to perform a meta‐analysis on five risk factors for BE or EAC which will be outlined below.

**TABLE 1:** Study characteristics.

Study, year (reference)	Study setting, country	Study design	Risk factors assessed	Number of case patients/control patients	Mean age of case patients/control patients	Control group
Anderson et al. 2009 [21]	Patient database, Northern Ireland and the Republic of Ireland	Case–control	Gender Smoking	BE (224) EAC (227) Control (260)	BE (62.4) EAC (64.2) Control (63)	Healthy
Asreah et al. 2021 [22]	Teaching Hospital, Iraq	Cross‐sectional	Gender Smoking	BE (13) Control (126)	BE (55.1) Control (38.7)	Endoscopic
Avidan et al. 2002 [72]	Ambulatory clinic, US	Case–control	Smoking Alcohol	BE (1189) EAC (131) Control (2170)	BE (61) Control (59)	Endoscopic
Baik et al. 2017 [73]	Ambulatory clinic, US	Case–control	Smoking	BE (31) Control (27)	BE (62.5) Control (52.6)	Endoscopic
Bakr et al. 2018 [23]	Integrated heath health services delivery organization, US	Case–control	Gender Reflux	BE (320) Control (317)	BE (62) Control (62)	Healthy
Bu et al. 2006 [24]	Hospital, US	Case–control	Gender	BE (174) Control (274)	Not specified	Endoscopic
Chacaltana et al. 2009 [25]	Hospital, Peru	Nested case–control	Gender Smoking Alcohol	BE (11) Control (911)	BE 52.2 Control (50.5)	Healthy
Chak et al. 2002 [26]	Hospital, US	Case–control	Gender Smoking Reflux Alcohol	BE (35) EAC (16) Control (106)	Not specified	Endoscopic
Chen et al. 2016 [27]	Hospital, Taiwan	Case–control	Gender Smoking Alcohol	BE (161) Control (644)	BE (53.8) Control (53.7)	Endoscopic
Chen et al. 2019 [28]	Endoscopy clinic, Taiwan	Case–control	Gender Smoking Alcohol	BE (89) Control (3296)	BE (55.6) Control (51.2)	Endoscopic
Corley et al. 2006 [29]	Integrated health services delivery organization, US	Nested case–control	Gender Obesity	BE (421) Control (842)	Not specified	Healthy
Corley et al. 2007 [30]	Integrated health services delivery organization, US	Nested Case–control	Gender Obesity Smoking Reflux	BE (320) Control (317)	Not specified	Healthy
De Ceglie et al. 2011 [31]	Endoscopy clinics, Italy	Case–control	Gender	BE (272) Control (517)	Not specified	Endoscopic
Dong et al. 2018 [32]	Patient database, United Kingdom	Case–control	Gender Smoking	BE (3288) EAC (2511) Control (2177)	BE (62.9) EAC (64.5) Control (61.7)	Healthy
Drahos et al. 2015 [33]	SEER‐Medicare database, US	Case–control	Gender Smoking	BE (2198) Control (6594)	BE (76.6) Control (76.7)	Healthy
Drahos et al. 2016 [34]	Electronic medical record database, United Kingdom	Case–control	Gender Smoking	BE (10215) Control (50167)	BE (64.0) Control (63.7)	Healthy
Edelstein et al. 2007 [35]	Endoscopy clinics, US	Case–control	Gender Obesity	BE (193) Control (211)	Not specified	Healthy
El‐Serag et al. 2005 [36]	Endoscopy clinic, US	Case–control	Gender Obesity	BE (36) Control (93)	BE (64) Control (63)	Abdominal computerized tomography patients
El‐Serag et al. 2014 [37]	Endoscopy clinic, US	Case–control	Gender Obesity Smoking Reflux Alcohol	BE (173) Control (172)	BE (63.1) Control (63.1)	Endoscopic
Filiberti et al. 2021 [38]	Endoscopy clinics, Italy	Case–control	Gender Smoking Reflux	BE (320) Control (606)	Not specified	Endoscopic
Iyer et al. 2013 [39]	Patient database, United Kingdom	Case–control	Gender Obesity Smoking	BE (14245) Control (70361)	BE (64.0) Control (64.0)	Healthy
Jiao et al. 2013 [40]	Hospital database, US	Case–control	Gender Smoking Reflux, Alcohol	BE (151) Control (777)	Not specified	Endoscopic
Johansson et al. 2007 [41]	Hospital database, Sweeden	Case–control	Gender, smoking	BE (21) Control (160)	BE (60.3) Control (61.8)	Healthy
Kendall et al. 2013 [42]	Patient database, Australia	Nested case–control	Gender Obesity Smoking Alcohol	BE (237) Control (247)	Not specified	Healthy
Kendall et al. 2014 [43]	Patient database, Australia	Nested case–control	Gender BMI, Smoking, Reflux, Alcohol	BE (235) Control (244)	Not specified	Healthy
Kendall et al. 2016 [45]	International patient database, Australia	Case–control	Gender, Smoking Reflux	BE (1559) Control (2557)	BE (60) Control (58.2)	Healthy
Kendall et al. 2020 [44]	Patient database, Australia	Nested case–control	Gender, Smoking Reflux	BE (227) Control (241)	Not specified	Healthy
Khalaf et al. 2014 [46]	Endoscopy clinic, US	Case–control	Gender Obesity Smoking Reflux	BE (323) Control (1849)	BE (61.6) Control (60.1)	Endoscopic and healthy
Kramer et al. 2013 [47]	Endoscopy clinic, US	Case‐ control	Gender Obesity	BE (237) Control (1500)	BE (62.1) Control (61.4)	Endoscopic
Kubo et al. 2009 [48]	Integrated health services delivery organization, US	Case–control	Gender Smoking, Alcohol	BE (320) Control (317)	Not specified	Healthy
Kubota et al. 2022 [49]	Endoscopy clinic, Japan	Case–control	Gender Smoking Reflux Alcohol	BE (2279) Control (7843)	Not specified	Endoscopic
Ladanchuk et al. 2010 [50]	Patient database, Republic of Ireland	Case–control	Gender Smoking, Reflux, Alcohol	BE (224) EAC (227) Control (260)	BE (62.4) EAC (64.2) Control (63)	Healthy
Lam et al. 2018 [51]	General practice database, United Kingdom	Case–control	Gender Obesity Smoking	BE (203) Control (23907)	BE (60.3) Control (59.0)	Healthy
Leggett et al. 2013 [52]	Patient database, US	Case–control	Gender Obesity Smoking Alcohol	BE (103) Control (103)	BE (60) Control (60)	Healthy
Lin et al. 2013 [53]	Endoscopy clinic, US	Case–control	Gender Smoking Alcohol	BE (285) Control (1121)	BE (61.5) Control (59.7)	Endoscopic
Lindam et al. 2013 [54]	Patient database, Australia	Nested case–control	Gender Obesity Smoking Reflux	BE (237) Control (247)	Not specified	Healthy
Mulholland et al. 2011 [55]	Patient database, Northern Ireland and the Republic of Ireland	Case–control	Gender Smoking	BE (212) EAC (218) Control (252)	BE (62.3) EAC (64.2) Control (62.8)	Healthy
Omer et al. 2012 [56]	Hospital endoscopy unit, US	Case–control	Gender BMI	BE (434) Control (434)	BE (60.8) Control (62.5)	Endoscopic
Park et al. 2009 [57]	40 hospital endoscopy units, Korea	Case–control	Gender smoking	BE (215) Control (23350)	Not specified	Endoscopic
Peters et al. 2021 [58]	Three Hospital endoscopy units, The Netherlands	Case–control	Gender Smoking Reflux	BE (480) Control (422)	BE (65.8) Control (62.5)	Healthy
Petrick et al. 2019 [59]	International patient database, US	Nested Case–control	Gender Smoking	BE (1728) BE Control (2830) EAC (2309) EAC Control (11841)	BE (60.5) Control (60.5)	Healthy
Schmidt et al. 2020 [60]	Disease registry, Germany	Case–control	Gender Smoking	BE (587) Control (1976)	BE (63.4) Control (59.3)	Healthy
Schneider et al. 2015 [61]	Integrated health services delivery organization, US	Case–control	Gender, smoking	BE (320) Control (317)	BE (61.9) Control (62.5)	Healthy
Smith et al. 2005 [62]	Patient database, Australia	Case–control	Gender	BE (167) Control (261)	BE (63) Control (63)	Healthy
Smith et al. 2009 [69]	Patient database, Australia	Case–control	Obesity Smoking Reflux Alcohol	BE (285) Control (644)	BE (58.2) Control (57.9)	Healthy
Steevens et al. 2011 [70]	National patient registry, Netherlands	Case‐cohort	Smoking	BE (870) Cohort (3866)	BE (61) Cohort (61)	Healthy
Thrift et al. 2012 [63]	Patient database, Australia	Case–control	Gender Obesity Smoking Reflux	BE (312) Control (398)	Not specified	Healthy
Thrift et al. 2015 [74]	Primary care clinics and endoscopy units, US	Nested Case–control	Obesity Smoking	BE (244) Control (824)	BE (61.6) Control (61.2)	Healthy and endoscopic
Vaughan et al. 1995 [67]	Patient registry, US	Case–control	Gender Smoking	EAC (298) Control (724)	EAC (61.1) Control (59.8)	Healthy
Veugelers et al. 2006 [64]	Hospital database, Canada	Case–control	Gender Obesity Smoking	BE (130) EAC (57) Control (102)	BE (59) EAC (67) Control (57)	Healthy
Vogtmann et al. 2015 [68]	Integrated health services delivery organization, US	Case–control	Gender Smoking Alcohol	EAC (626) Control (49886)	EAC (66.5) Control (67.2)	Healthy
Wang et al. 2022 [65]	Patient database, Australia	Nested case–control	Gender Smoking	BE (169) Control (685)	Not specified	Healthy
Xie et al. 2020 [66]	International database and registries, Australia, Europe and North America	Case–control	Gender Smoking	BE (3.247) EAC (2488) Control (2127) EAC (244) Control (244)	Not specified	Healthy
Xie et al. 2020 [71]	Patient database and cancer registry, Norway	Nested Case–control	Obesity Smoking	EAC (244) Control (244)	Not specified	Healthy

### 3.1 Gender

**FIGURE 2:**
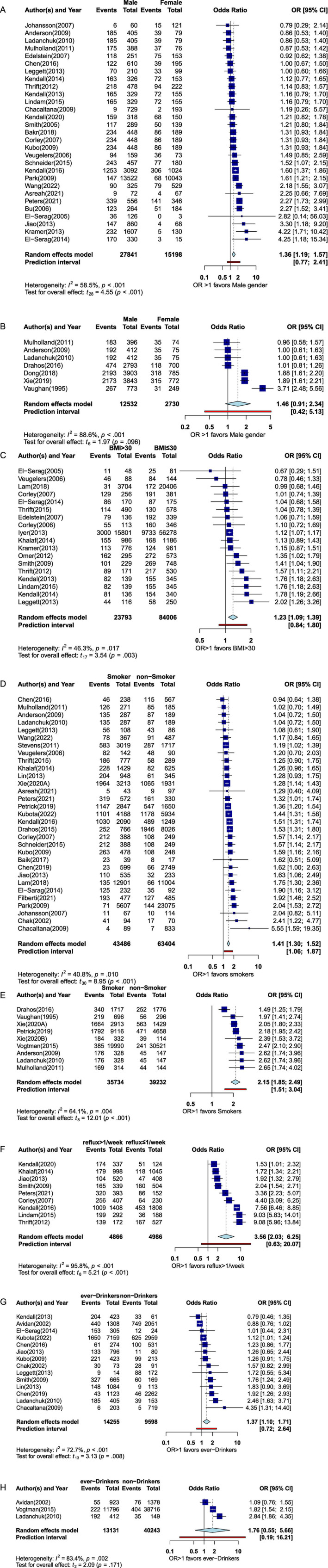
Forest plots for outcomes data. (A) Forest plot of pooled OR for male gender as a risk factor for being diagnosed with BE. (B) Forest plot of the pooled OR for male sex as a risk factor for being diagnosed with EAC. (C) Forest plot of the pooled OR for obesity as a risk factor with being diagnosed BE. (D) Forest plot of pooled OR for tobacco smoking as a risk factor for being diagnosed with BE. (E) Forest plot of pooled OR for tobacco smoking as a risk factor for being diagnosed with EAC. (F) Forest plot of pooled OR for reflux more than once weekly as a risk factor for being diagnosed with BE. (G) Forest plot of pooled OR for alcohol use as a risk factor for being diagnosed with BE. (H) Forest plot of pooled OR for alcohol use as a risk factor for being diagnosed with EAC.

Forty‐six studies meeting our inclusion criteria, with 32,242 men and 15,131 women, reported data on gender for BE patients [21, 22, 23, 24, 25, 26, 27, 28, 29, 30, 31, 32, 33, 34, 35, 36, 37, 38, 39, 40, 41, 42, 43, 44, 45, 46, 47, 48, 49, 50, 51, 52, 53, 54, 55, 56, 57, 58, 59, 60, 61, 62, 63, 64, 65, 66]. Upon examination of the funnel plots, we identified 17 studeis as outliers [26, 28, 29, 31, 32, 33, 34, 38, 39, 46, 48, 51, 53, 56, 59, 60, 66] which were removed using leave‐one‐out sensitivity analysis (Figure S1A,B). Meta‐analysis of the remaining studies showed a significant pooled OR favoring male gender in BE patients (1.36; 95% CI, 1.19–1.57; *p* < 0.001) (Figure 2A).

Nine included studies, including 8278 men and 1218 women, assessed gender in EAC patients [21, 32, 34, 50, 55, 59, 66, 67, 68]. Two studies [59, 67] were identified as outliers on the funnel plots and removed by employing leave‐one‐out sensitivity analysis (Figure S1C,D). This meta‐analysis resulted in a nonsignificant pooled OR favoring male gender in EAC patients (1.46; 95% CI, 0.91–2.34; *p* = 0.096) (Figure 2B).

### 3.2 Obesity

Data regarding body mass index (BMI) in kg/m^2^ was found in 18 of our included studies [29, 30, 35, 36, 37, 39, 42, 43, 46, 47, 51, 52, 54, 56, 63, 64, 69, 70]. There was a total of 12,496 BE patients with a BMI less than 30 kg/m^2^ and 4537 with a BMI greater than 30 kg/m^2^. No outliers were detected, and the meta‐analysis showed a significant pooled OR discriminating for BMI greater than 30 kg/m^2^ in BE patients (1.23; 95% CI, 1.09–1.39; *p* = 0.003) (Figures 2C and S1E,F).

Only one of our included studies reported obesity data in EAC [71]. Thus, there was insufficient data to perform a meta‐analysis.

### 3.3 Tobacco Smoking

Forty‐two included studies contained data on smoking status for patients with BE [21, 22, 25, 26, 27, 28, 30, 32, 33, 34, 37, 38, 39, 40, 41, 42, 43, 44, 45, 46, 48, 49, 50, 51, 52, 53, 54, 55, 57, 58, 59, 60, 61, 63, 64, 65, 66, 69, 70, 72, 73, 74]. There were 24,698 ever‐smokers and 22,785 nonsmokers with BE. After performing sensitivity analysis, we excluded 11 studies identified as outliers [32, 34, 39, 42, 43, 44, 54, 60, 63, 69, 72] (Figure S1G,H). Meta‐analysis revealed a significant pooled OR for BE patients having ever been tobacco smokers (1.41; 95% CI, 1.30–1.52; *p* < 0.001) (Figure 2D).

Ten studies contained data for smoking in patients with EAC [21, 32, 34, 50, 55, 59, 66, 67, 68, 71]. Dong et al. [32] was found to be an outlier upon sensitivity analysis and removed (Figure S1I,J). This meta‐analysis resulted in a significant pooled OR favoring patients who have ever smoked tobacco being diagnosed with EAC (2.15; 95% CI, 1.85–2.49; *p* < 0.001) (Figure 2E).

### 3.4 Gastroesophageal Reflux (GER)

The frequency of GER symptoms was recorded for 13 of our included studies of patients with BE [23, 26, 30, 40, 44, 45, 46, 49, 50, 54, 58, 63, 69]. Patients with BE having reflux less than once weekly totaled 3124, while 3484 had reflux at least once weekly. Sensitivity analysis revealed [23, 26, 49, 50] to be outliers, and they were removed from our statistical analysis (Figure S1K,L). Results of this meta‐analysis revealed a significant pooled OR favoring patients having BE while reporting GER symptoms more than once weekly (3.56; 95% CI, 2.03–6.25; *p* = 0.004) (Figure 2F).

Only one study contained data on the frequency of GER symptoms in patients with EAC; therefore, it was not possible to conduct a meta‐analysis [50].

### 3.5 Alcohol Use

Data in BE patients who use alcohol was found in 15 of our included studies [25, 26, 27, 28, 37, 40, 42, 43, 48, 49, 50, 52, 53, 69, 72]. The total number of BE patients who were reported as nondrinkers of alcohol was 1981, whereas those who reported ever drinking were 3,868. Kendall et al. [43] were excluded after it was determined to be an outlier upon sensitivity analysis (Figure S1M,N). The meta‐analysis we performed showed a significant pooled OR favoring patients who have drunk alcohol for being diagnosed with BE (1.37; 95% CI, 1.10–1.71; *p* = 0.008) (Figure 2G).

Only three of our included studies contained data on alcohol drinking in patients with EAC [50, 68, 72]. The nondrinking EAC patients totaled 515, while the ever‐drinkers were 469. This meta‐analysis resulted in a non‐significant pooled OR favoring patients who were ever‐drinkers of alcohol for the diagnosis of EAC (1.76; 95% CI, 0.55–5.66; *p* = 0.171) (Figures 2H and S1O,P).

### 3.6 Bariatric Surgery

Two studies met our inclusion criteria in examining the risk of developing BE after bariatric surgery in patients who had endoscopies prior to their surgery [75, 76]. However, there was not enough data to perform a meta‐analysis on this potential risk factor.

## 4 Discussion

Our systematic review has identified 54 studies which met inclusion criteria and were statistically analyzed for meta‐analyses. This meta‐analysis contained a wide scope of risk factors for the development of BE or EAC separately: gender; obesity; tobacco smoking; GER symptoms; and alcohol use. We identified significant associations for six pairings of risk factors and outcomes, all resulting in an OR which favored the clinical prediction of either BE or EAC. Five of these associations were with BE, and one was with EAC. The largest impact was seen in patients with GER symptoms at least once weekly and its association with the development of BE, resulting in a significant pooled OR (3.56; 95% CI; 2.03–6.25; *p* = 0.004) (Figure 2). The other statistically significant associations in descending order of the magnitude are: tobacco smoking favors the development of EAC (OR, 2.15; 95% CI, 1.85–2.43; *p* < 0.001) tobacco smoking increases the risk of attaining BE (OR, 1.41; 95% CI, 1.30–1.51; *p* < 0.001); alcohol use was shown to predict the diagnosis of BE (OR, 1.37; 95% CI 1.10–1.71; *p* = 0.008); male gender favors being diagnosed with BE (OR, 1.36; 95% CI, 1.19–1.57; *p* < 0.001); and lastly obesity (BMI > 30 kg/m^2^) increased the probability of being diagnosed with BE (OR, 1.23; 95% CI, 1.09–1.39; *p* = 0.003) (Figure 2). To our knowledge, these data are derived from the largest meta‐analysis yet to be conducted on screening for BE or EAC.

Our findings regarding the magnitude of weekly GER symptoms as a risk factor for the development of BE were also supported by an earlier systematic review and meta‐analysis by Qumseya et al., who demonstrated a 0.8% prevalence of BE compared with a 3.0% prevalence in patients with GERD [77]. The presence of GERD plus any other risk factor corresponded with a BE prevalence of 12.2% in this study [77]. Two other systematic reviews with meta‐analyses have also reported a higher BE prevalence in patients with GER symptoms and in patients with weekly GER symptoms, respectively [78, 79]. The later study reported a smaller effect of weekly GER symptoms on the prevalence of BE (OR 1.67) compared with our study (OR 1.67; 95% CI, 1.30—2.15) [79] Our study, along with data from these studies above, should draw more attention to GER symptoms and GERD as stronger risks for the development of BE than previously recognized [9]. As mentioned earlier, there are five organizations in the United States and Europe that have published guidelines for BE screening and diagnosis based on risk factors [9, 10, 11, 12]. Recognizing the inequality of all the effects of the known risk factors for developing BE, we propose weighing these risk factors based on their individual contribution.

The hypothesis for BE screening is based on the metaplasia‐dysplasia‐carcinoma progression paradigm. Based on this paradigm, endoscopic BE screening could identify patients who may benefit from surveillance and treatment programs which may modify the risk of progression to EAC [9]. Support for this hypothesis has been shown in patients diagnosed with BE correlated with better overall survival, lower all‐cause mortality and finding EAC at earlier stages [8, 80]. The finding of less than 7.3% of EAC patients were known to have BE prior to their cancer diagnosis suggests that either screening guidelines are not being implemented or require further evaluation [8, 81]. Given the less than 20% survival of EAC once symptoms occur, further examination of screening protocols deserves our attention [81]. Screening the general population for BE has not shown to be cost effective due to the low prevalence of the disease in the general population. However, Benaglia et al., have shown that a screening model for 50‐year‐old patients with GER symptoms using a non‐endoscopic cell sampling device is cost effective and would reduce mortality form EAC compared with no screening [82]. Other models have shown cost effectiveness in screening for BE in 60‐year‐old patients with GERD by a minimally invasive cell sampling device followed by endoscopy [83]. We support the employment of similar screening strategies to augment the diagnosis of BE, especially knowing the majority of patients with EAC are not identified for BE surveillance programs [9].

The most obvious limitation to our paper is that all our data were derived from observational studies. All of our included studies were characterized as observational (Table 1) and according to the Grading of Recommendations, Assessment, Development and Evaluation, our derived data are deemed low‐quality evidence [18]. We mitigated the risk of bias found in observational studies by limiting our included studies to only those which had control groups for comparison to case patients. Regarding our included studies, only 22 had a low risk of bias (Table 2). We identified 24 of our included studies to have bias in either the domain of selection or comparability due to either the use of endoscopic controls rather than healthy controls or lack of comparability between case and control patients, respectively (Table 2). Bias due to confounding is possible for race and the collection of data through insurance registries. Both BE and EAC is more common in persons of white race, and Kendall et al. [45] only included non‐Hispanic white patients. We agree with the American College of Gastroenterology and did not account for race as a biological variable but rather as a social construct that stratifies patients [9]. Data from six studies were obtained from the same insurance registry (Kaiser Permanente) and they all deemed their study populations to be demographically representative of the Northern California census population [23, 29, 30, 48, 61, 68]. Publication bias was mitigated by using leave‐one‐out sensitivity analysis when the effect size was outside the confidence intervals of the pooled effect size (Figure S1). Our study design was to identify risk factors for BE or EAC independent of prior diagnoses: therefore, we did not report on hiatal hernia, which has been reported as a well‐established risk factor for developing BE. Lastly, cost effectiveness for BE diagnosis using risk factors needs further investigation.

**TABLE 2:** Risk of bias.

Study, year (reference)	1. Cases clearly defined	2. Consecutive or representative series of cases	3. Selection of controls	4. Definition of controls	5. Comparability of cases and controls	6. Same methods of ascertainment of case and controls
Anderson et al. 2009 [21]	1	1	1	1	1	1
Asreah et al. 2021 [22]	1	1	0	1	1	
Avidan et al. 2002 [72]	1	1	0	1	1	1
Baik et al. 2017 [73]	1	0	0	1	0	1
Bakr et al. 2018 [23]	1	1	1	1	1	1
Bu et al. 2006 [24]	1	1	0	1	1	1
Chacaltana et al. 2009 [25]	1	0	0	1	0	1
Chak et al. 2002 [26]	1	1	0	1	0	1
Chen et al. 2016 [27]	1	1	0	1	1	1
Chen et al. 2019 [28]	1	1	0	1	0	1
Corley et al. 2006 [29]	1	1	1	1	0	1
Corley et al. 2007 [30]	1	1	1	1	0	1
De Ceglie et al. 2011 [31]	1	1	1	1	0	1
Dong et al. 2018 [32]	1	1	1	1	1	1
Drahos et al. 2015 [33]						
	1	1	1	1	1	1
Drahos et al. 2016 [34]	1	1	1	1	1	1
Edelstein et al. 2007 [35]	1	1	1	1	1	1
El‐Serag et al. 2005 [36]	1	1	1	1	1	1
El‐Serag et al. 2014 [37]	1	1	1	1	1	1
Filiberti et al. 2021 [38]	1	1	0	1	0	1
Iyer et al. 2013 [39]	1	1	1	1	1	1
Jiao et al. 2013 [40]	1	1	0	1	0	1
Johansson et al. 2007 [41]	1	1	1	1	1	1
Kendall et al. 2013 [42]	1	1	1	1	0	1
Kendall et al. 2014 [43]	1	1	1	1	0	1
Kendall et al. 2016 [45]	1	0	1	1	1	1
Kendall et al. 2020 [44]	1	1	1	1	0	1
Khalaf et al. 2014 [46]	1	1	0	1	0	1
Kramer et al. 2013 [47]	1	1	0	1	0	1
Kubo et al. 2009 [48]	1	1	0	1	1	1
Kubota et al. 2022 [49]	1	1	0	1	0	1
Ladanchuk et val., 2010 [50]	1	1	1	1	1	1
Lam et al. 2018 [51]	1	1	1	1	1	1
Leggett et al. 2013 [52]	1	1	1	1	1	1
Lin et al. 2013 [53]	1	1	0	1	1	1
Lindam et al. 2013 [54]	1	1	1	1	0	1
Mulholland et al. 2011 [55]	1	1	1	1	1	1
Omer et al. 2012 [56]	1	0	0	1	0	1
Park et al. 2009 [57]	1	1	0	1	0	1
Peters et al. 2021 [58]	1	1	1	1	0	1
Petrick et al. 2019 [59]	1	1	1	1	1	1
Schmidt et al. 2020 [60]	1	1	1	1	0	0
Schneider et al. 2015 [61]	1	1	1	1	1	1
Smith et al. 2005 [62]	1	1	1	1	1	1
Smith et al. 2009 [69]	1	1	1	1	1	1
Steevens et al. 2011 [70]	1	1	1	1	1	1
Thrift et al. 2012 [63]		1	1	1	0	1
Thrift et al. 2015 [74]	1	1	0	1	1	1
Vaughan et al. 1995 [67]	1	1	1	1	1	1
Veugelers et al. 2006 [64]	1	1	1	1	1	1
Vogtman et al. 2015 [68]	1	1	1	1	1	1
Wang et al. 2022 [65]	1	1	1	1	0	1
Xie et al. 2020 [66]	1	1	1	1	0	1
Xie et al. 2020 [71]	1	1	1	1	0	1

*Note:* Cases were independently assigned/validated: 1 for yes, 0 for record linkage based on self‐reports or no description; Consecutive or obviously representative series of cases: 1 for yes, 0 for selection bias; Selection of community controls: 1 for yes, 0 for Hospital controls or endoscopy controls; Definition of controls: 1 for healthy, 0 for no definition of source; Comparable cases and controls: 1 for yes, 0 for no; Ascertainment of results blinded: 1 for yes, 0 for no; Consistent method of ascertainment for cases and controls: 1 your yes, 0 for no. Color key: Green, low risk of bias; Yellow, moderate risk of bias; Red, high risk of bias.

In conclusion, our meta‐analysis provides a comprehensive database on risk factors and clinical indicators to help identify the patients who are at the highest risk of developing BE and could benefit from a diagnostic evaluation. We present evidence supporting a strong relationship with patients who suffer from symptoms of weekly gastroesophageal reflux and the development of BE, the only known pathological precursor to EAC. Until further evidence supports the diagnostic accuracy of less invasive endoscopic procedures or biologic markers for screening, we advocate for increased awareness of BE risk factors by primary care physicians to increase endoscopic evaluations. The opportunity for enhancing the low rates for early diagnosis of BE appears to be a current pathway in improving the unfavorable epidemiology of EAC.

## Author Contributions

Conception and design of the study: Kais Anntonios, Eliot Berenstein, Patricia McNally, Sichao Wang, Richard A. Shellenberger. Aquistion of data: All authors. Analysis and intrepretation of data: Daniel Aintabi, Claudia Villarreal Carrillo, Sichao Wang and Richard A. Shellenberger. Drafting and revising the manuscript: All authors. Final approval of the the version submitted for publictaion: All authors.

## Conflicts of Interest

The authors declare no conflicts of interest.

## Supporting information


**Supporting Information S1.** Study protocol.

**Figure S1.** Funnel plots.

## Data Availability

All raw data used for the statsitcal analysis to support the conclusions of this study are available upon request to the corersponding author.
