# Supplementary material for: Risk Factors for the Development of Barrett's Esophagus and Esophageal Adenocarcinoma: A Systematic Review and Meta‐Analysis
Source: Cancer Rep (Hoboken). 2025 Mar 4;8(3):e70168. doi: 10.1002/cnr2.70168 (PMC11880629; doi:10.1002/cnr2.70168)
Supplement: Supplementary file 1 — Supporting Information S1. Study protocol. [file CNR2-8-e70168-s001.docx]

**Supplement 1. Study Protocol**

**Title:** Identifying clinically important risk factors for the development of Barrett’s esophagus and esophageal adenocarcinoma. A systematic review and meta-analysis

**Authors**

Corresponding Author: Richard A Shellenberger, DO^1^

Coauthors: Kais Antonios, MD

Daniel Aintabi, MD

Elliot Berinstein, MD

Patricia McNally, MD

Priyata Dutta, MD

Nicholas Sampson, BS

Sichao Wang, Ph.D.

Claudia Villarreal Carrillo, MD

Brahm Singh, DO

Marjan Haider, MD

**Introduction and PICO question:**

Esophageal adenocarcinoma (EAC) has a rising incidence rate and mortality rate in the US, several European countries as well as Australia over the past three decades.^1^ The prognosis of EAC is not very favorable, with only a 20% five-year survival in the US. Like most cancers, EAC has a better prognosis if detected at earlier stages. There are no known screening guidelines in the US for Barrett’s or for early detection of EAC.

Known risk factors for the development of EAC include chronic gastroesophageal reflux, obesity, and male sex. Barrett’s esophagus (BE) is the most widely established precursor to EAC We formed the study question following a guide which identifies these characteristics: patients or problem, and intervention, comparison group, outcomes, and study design (PICOS).

**PICOS question**: Are data available which identifies patients at high risk for the development of Barrett’s esophagus or esophageal adenocarcinoma that may be useful to support endoscopic screening guidelines.

**Protocol**

This systematic review was registered through PROSPERO (ID: ). We followed the Preferred Reporting Items for Systematic Reviews and Meta-Analysis (PRISMA) Guideline and have a PRISMA checklist.

**Search strategy:**

Using common indexing practices, we searched repeatedly using key words and medical subject headings to capture potentially relevant publications or our database. PubMed.gov, Ovid Medline, Embase, and the Cochrane Library from the inception date until March 1, 2023, without language restrictions. Searches were performed employing the following keywords: risk factors; Barrett’s esophagus; esophageal adenocarcinoma; screening; early detection; patient characteristics; comorbidities; diagnosis; predictors; patient factors; population; and screening guidelines.

**Key words and searches :**

Searches were performed employing the following keywords: risk factors; Barrett’s esophagus; esophageal adenocarcinoma; screening; early detection; patient characteristics; comorbidities; diagnosis; predictors; patient factors; population; and screening guidelines.

**Embase searches:**

Search 1: Barrett esophagus AND early detection - 1065 articles

Search 2: Barrett esophagus AND When AND to screen for - 42 articles

Search 3: Barrett esophagus AND at risk AND population - 868 articles

Search 4: Barrett esophagus And Screening guidelines And GERD - 79 Articles

**PubMed searches:**

Search 1: Risk factors AND Barrett’s esophagus - 29 articles

Search 2: Screening AND Early Detection of Barrett’s Esophagus OR Esophageal Adenocarcinoma - 1035 articles

Search 3: Patient characteristics OR Comorbidities AND risk of developing Barrett’s esophagus or Esophageal adenocarcinoma – 1012 articles.

**OVID Medline Searches:**

Barrett’s esophagus AND risk factors OR patient characteristics AND diagnosis OR esophageal adenocarcinoma – 604 articles

**Cochrane library searches:**

Barrett’s Esophagus OR Barrett’s OR esophagus OR Barrett’s Oesophagus AND risk factors for diagnosis OR development - 720 articles

**Study selection and inclusion/exclusion criteria**

Two independent investigators ( and RS) complied a reference list that was uploaded into a file management software (Covidence). Duplicates were removed and the two investigators separately screened titles and abstracts for eligibility criteria. Studies possibly meeting inclusion criteria were saved for full text review after a consensus agreement by both investigators.

Randomized-control trials and prospective cohort observational studies were preferred due to lower risk of bias. We included studies which examined relationships of patient characteristics who developed either BE or EAC and compared them to healthy age and sex matched controls. Abstract only papers were excluded, as study design and methods of data acquisition may not be able to be evaluated and reconciled. Any laboratory test studied for the detection of BE or EAC had to be tests readily available to practicing physicians and generally approved for use. We excluded all studies which examine the role of genetic testing to screen for BE or EAC.  Subjects of all included studies had to be human.

**Data extraction and quality assessment**

Dual extraction was followed on all include studies to obtain data for statistical analysis. Study characteristics included: first author and date of study; study description; patient demographic data; Risk factors studied, and length of follow-up for each study Two investigators independently assessed the quality and risk of bias of all included studies using a modified Newcastle-Ottawa Quality Assessment Tool for the Observational, Cohort and Cross- Sectional Studies available from the National Institute of Health. The certainty of evidence was evaluated for each study by using the GRADE assessment.

**Data synthesis**

Meta-analysis will be considered whenever studies of similar design, participants and outcomes yield quantitative estimates that require pooling to increase precision. Since we will obtain literature from different patient populations, we anticipated that significant heterogeneity would exist. This heterogeneity can be attributed to multiple known and unknown factors. If possible, subgroup analyses will be conducted to explore the effect of each subgroup designation (covariate) on the observed associations. In particular, we will collect any reported data on age subgroups. If data were sufficient for quantitative analysis, interaction tests and meta-regression techniques will be used to investigate heterogeneity. We will adhere to the PRISMA guidelines when reporting the final findings of our study.

References

1. Coleman HG, Xie SH, Lagergren J. The Epidemiology of Esophageal Adenocarcinoma. *Gastroenterology.* 2018;154(2):390-405.
