# Supplementary material for: Risk Factors for the Development of Barrett's Esophagus and Esophageal Adenocarcinoma: A Systematic Review and Meta‐Analysis
Source: Cancer Rep (Hoboken). 2025 Mar 4;8(3):e70168. doi: 10.1002/cnr2.70168 (PMC11880629; doi:10.1002/cnr2.70168)
Supplement: Supplementary file 2 — Figure S1. Funnel plots. [file CNR2-8-e70168-s002.docx]

SuSupplement 2. Funnel plots


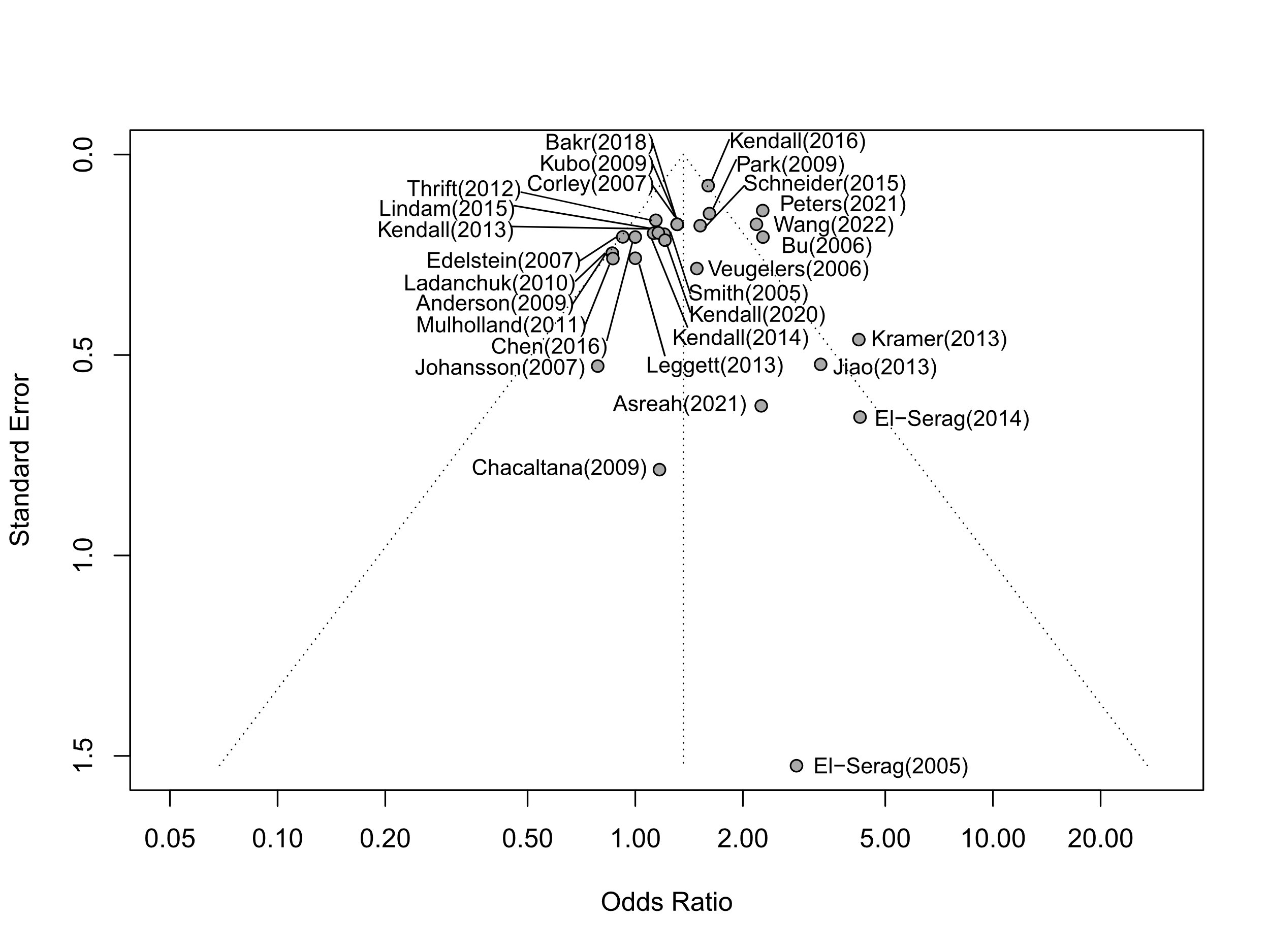


1. Funnel plot for Barrett’s esophagus gender versus control


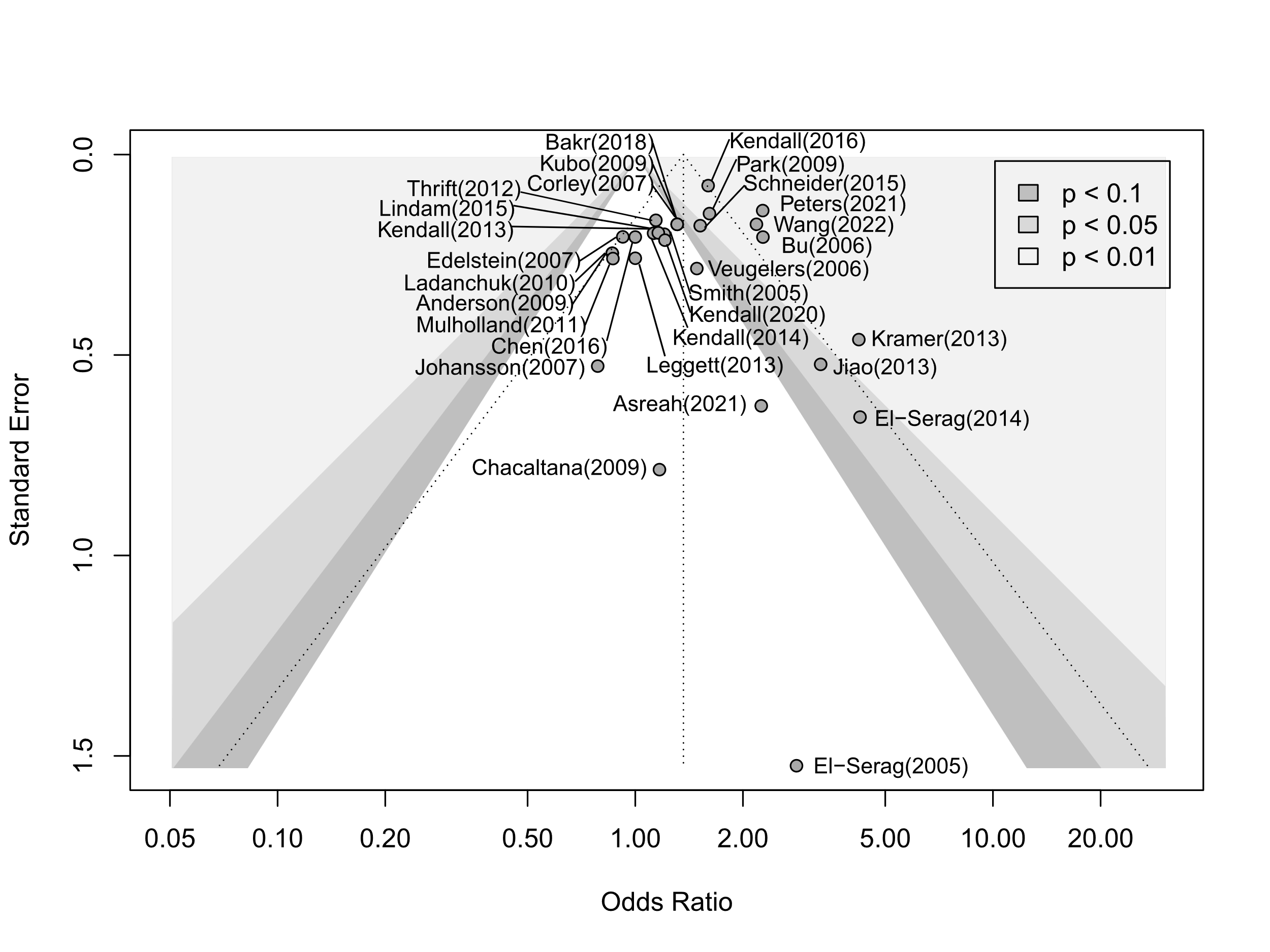


1. Contour enhanced funnel lot for Barrett’s esophagus gender versus control


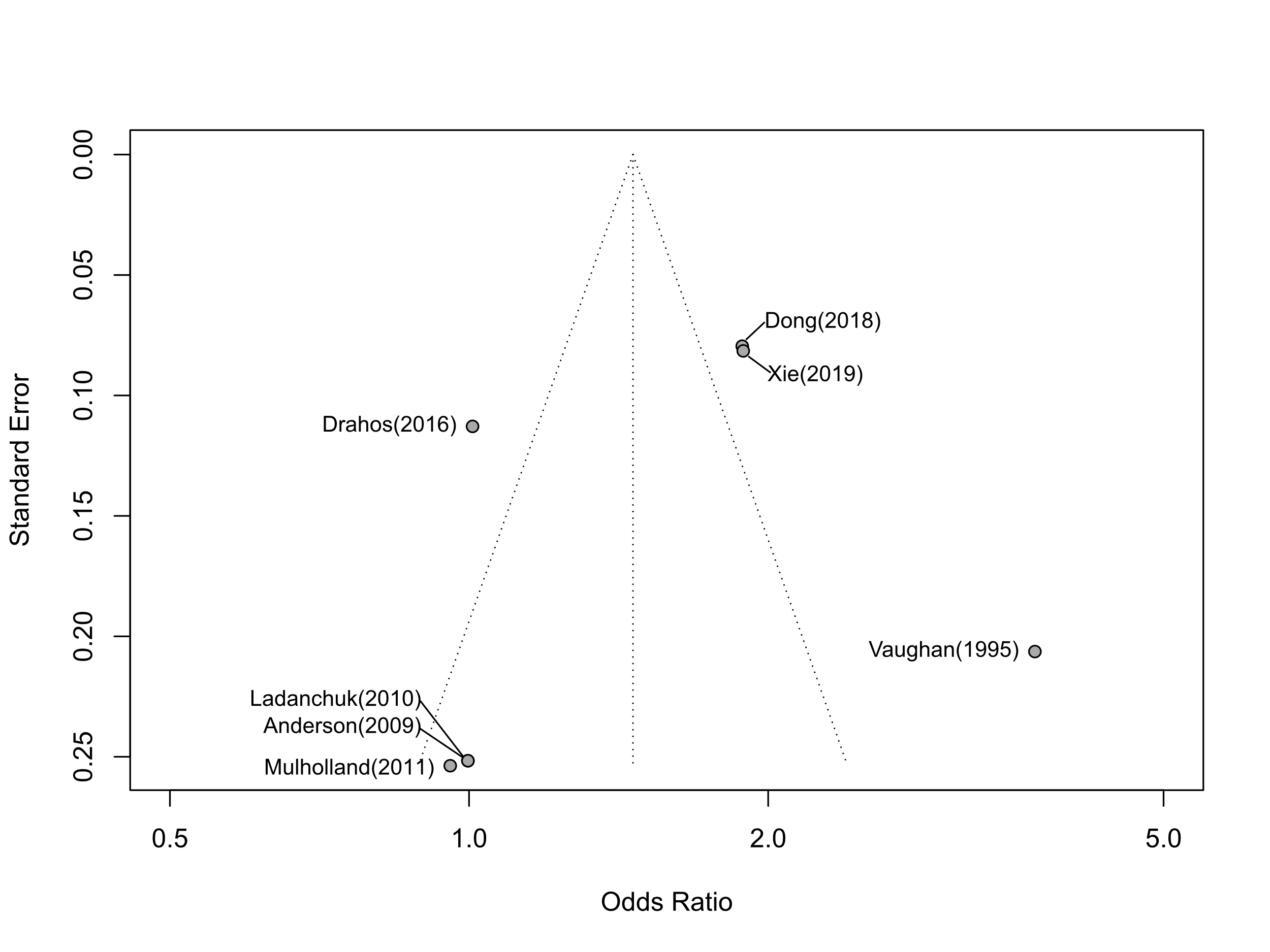


1. Funnel plot for esophageal adenocarcinoma gender versus control


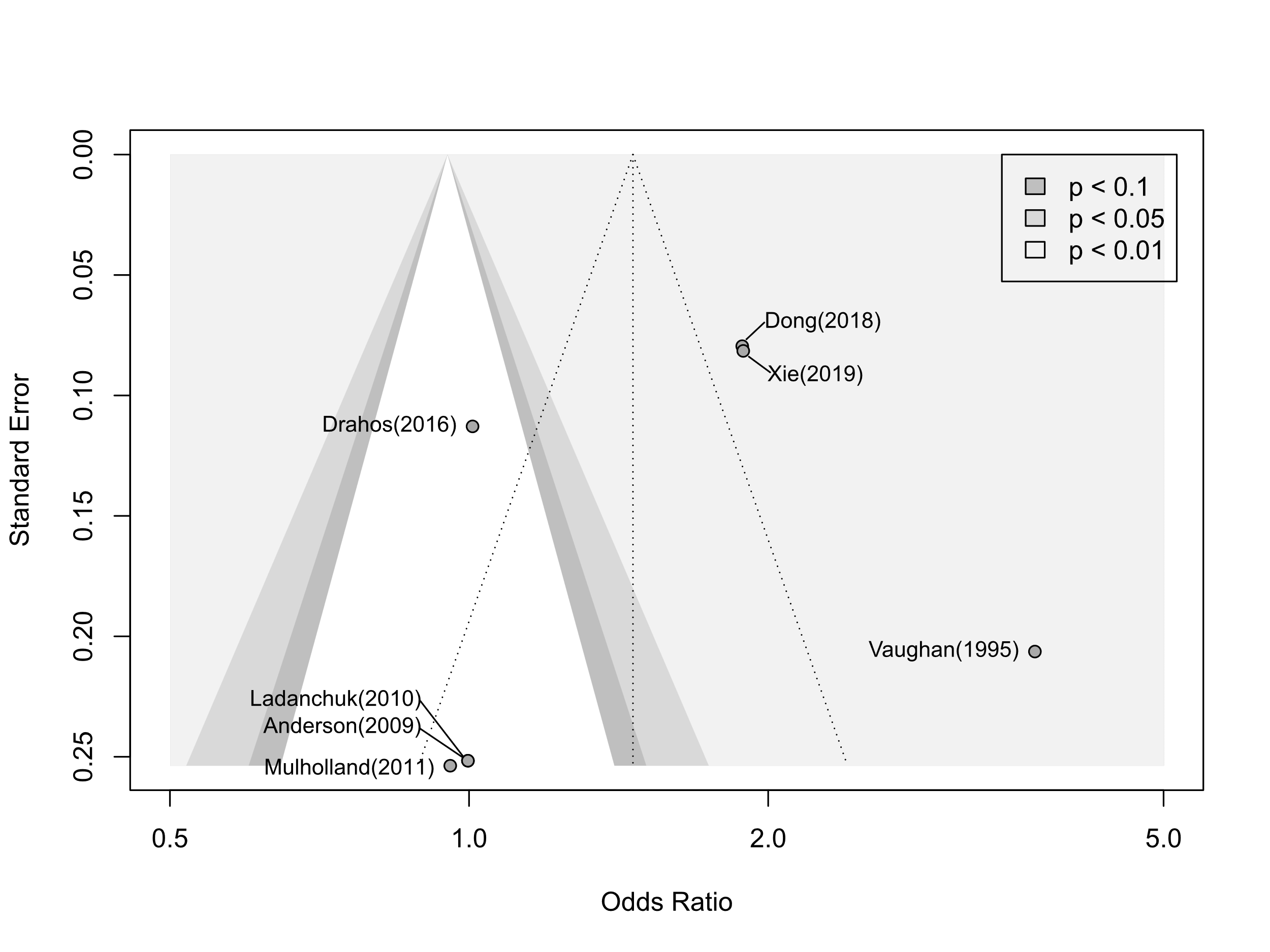


1. Contour enhanced funnel lot for esophageal adenocarcinoma gender versus control


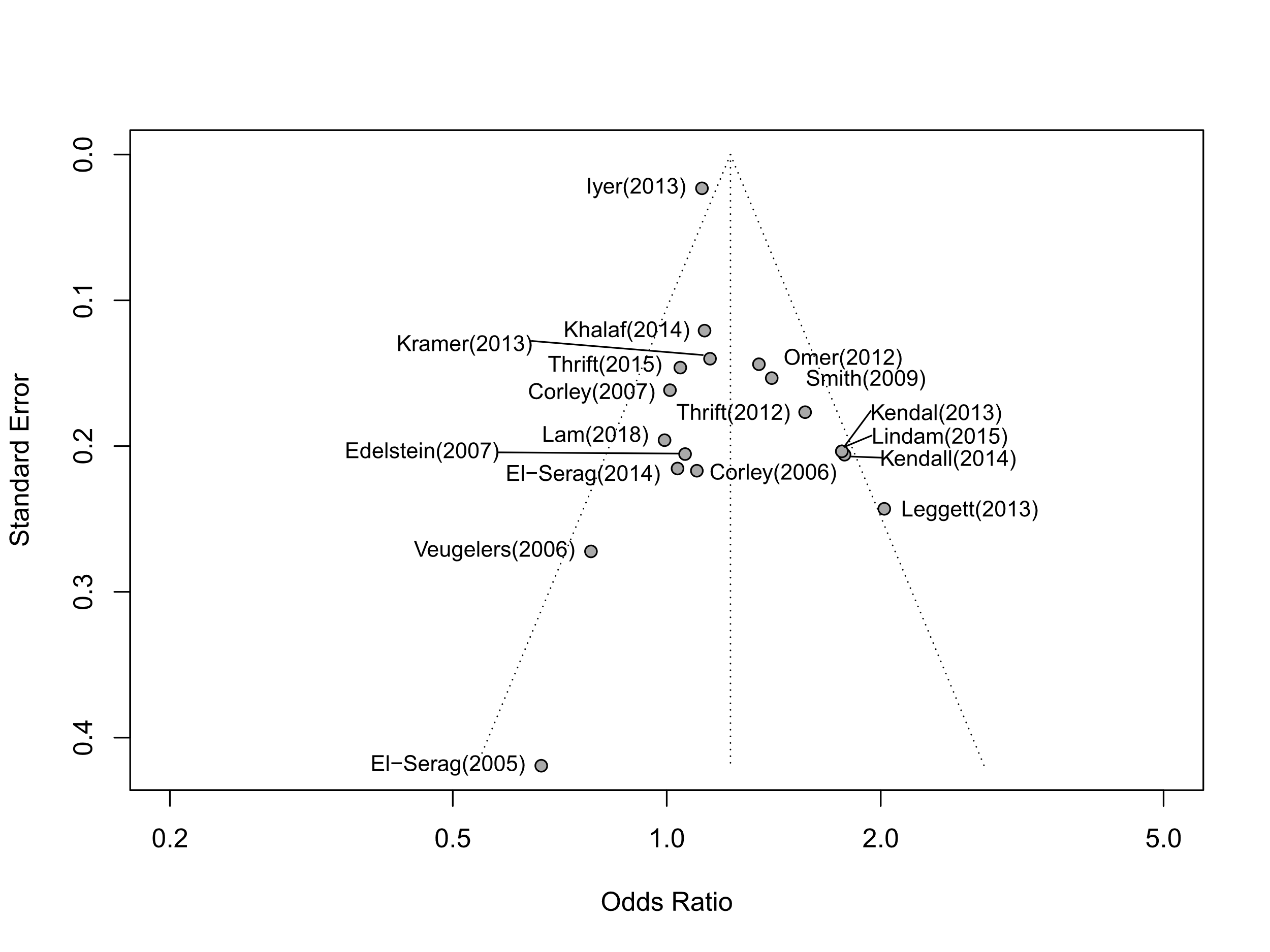


1. Funnel Plot for Barrett’s esophagus Obesity vs control


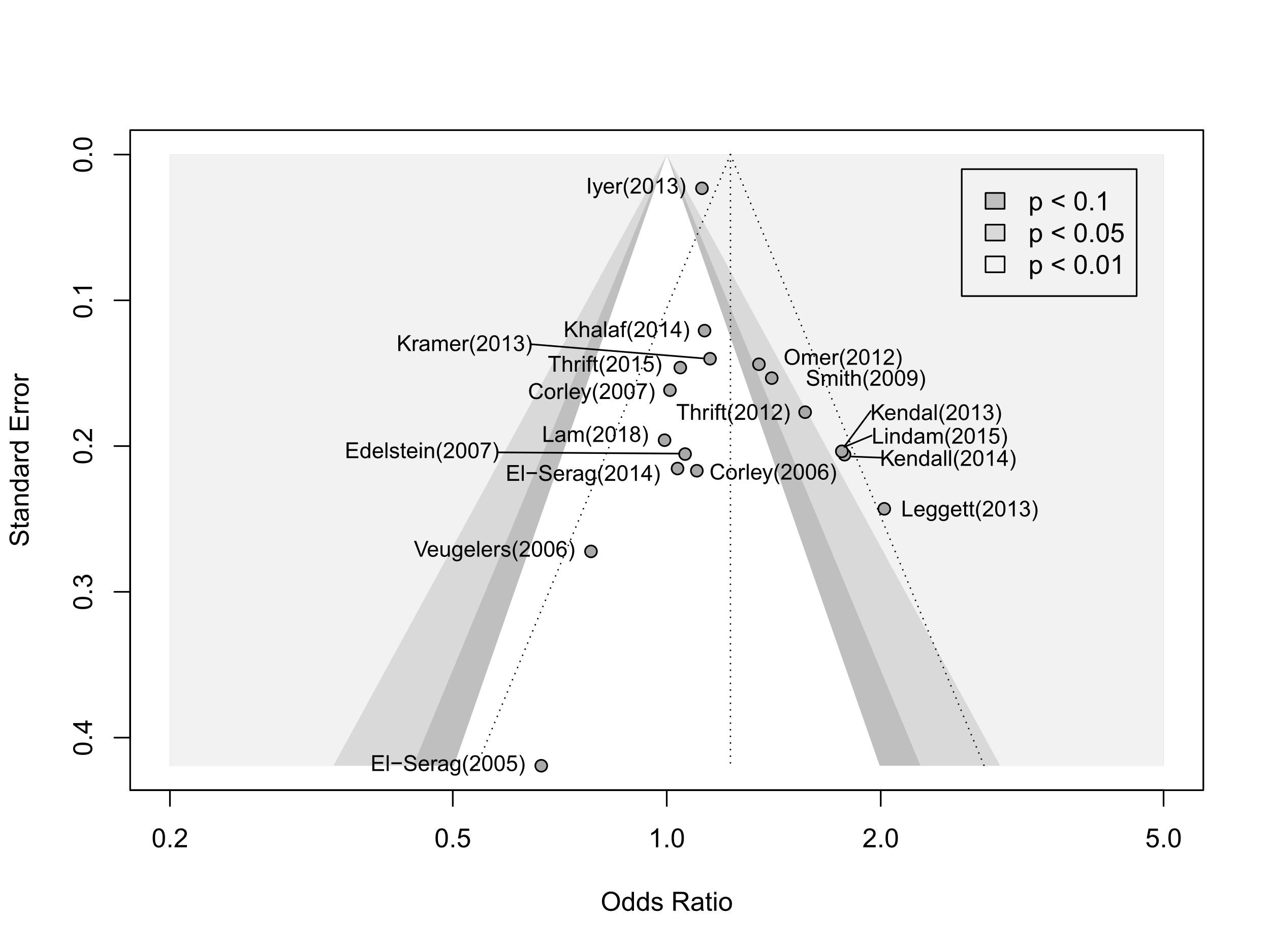


1. Contour-Enhanced funnel plot for Barrett’s esophagus obesity versus control


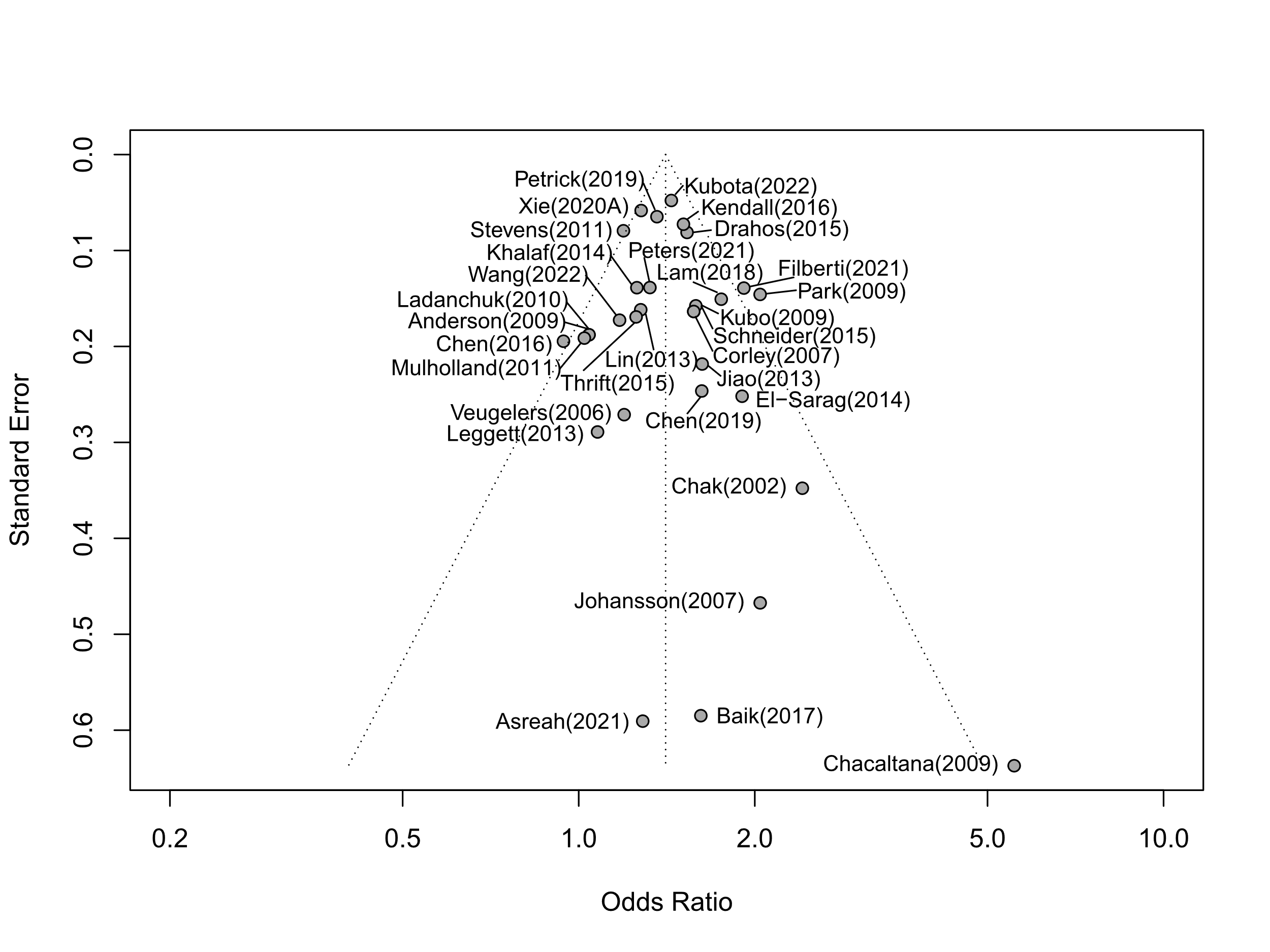


1. Funnel plot for Barrett’s esophagus tobacco use versus control


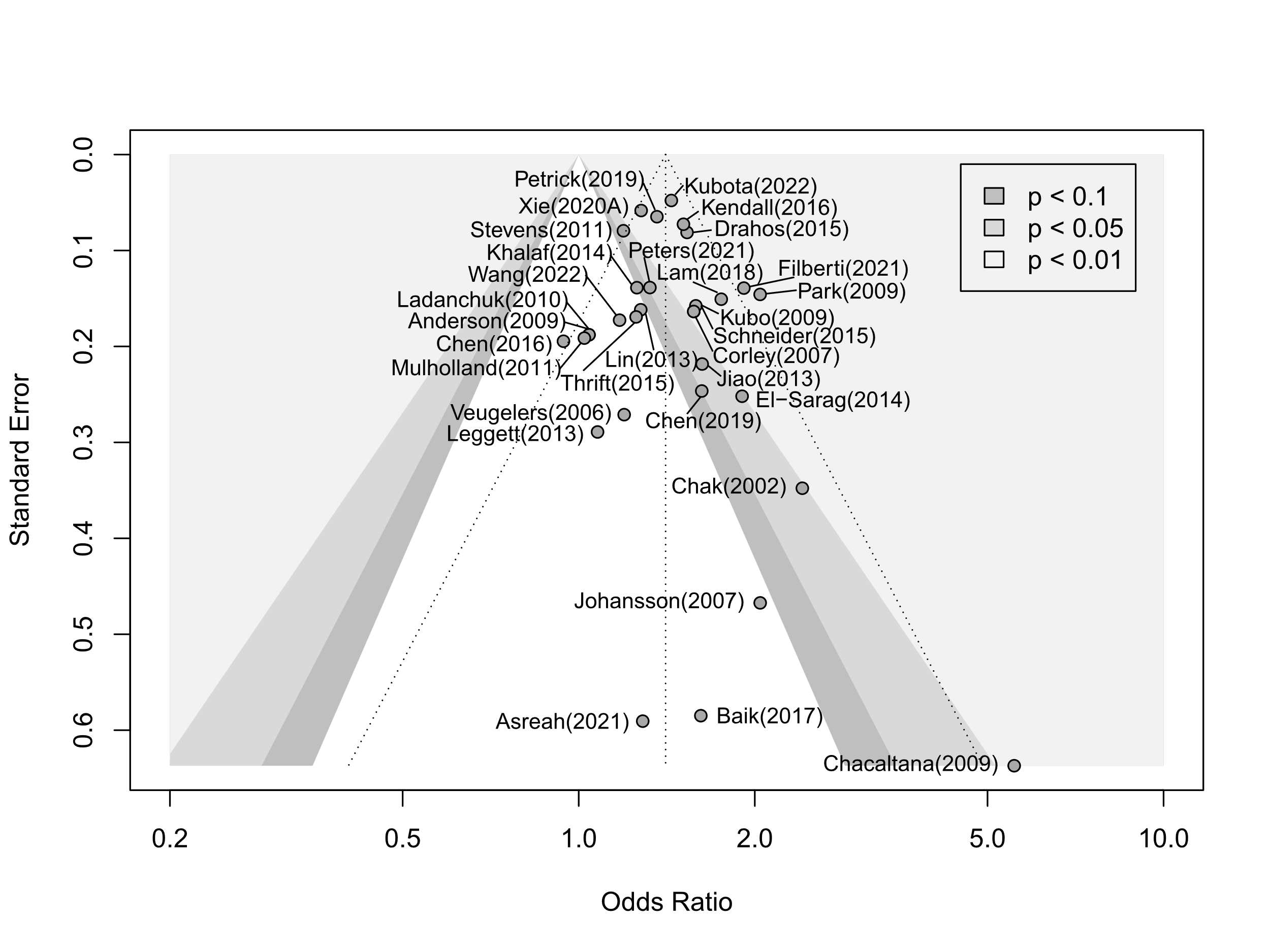


1. Contour-Enhanced funnel plot for Barrett’s Esophagus tobacco use versus control


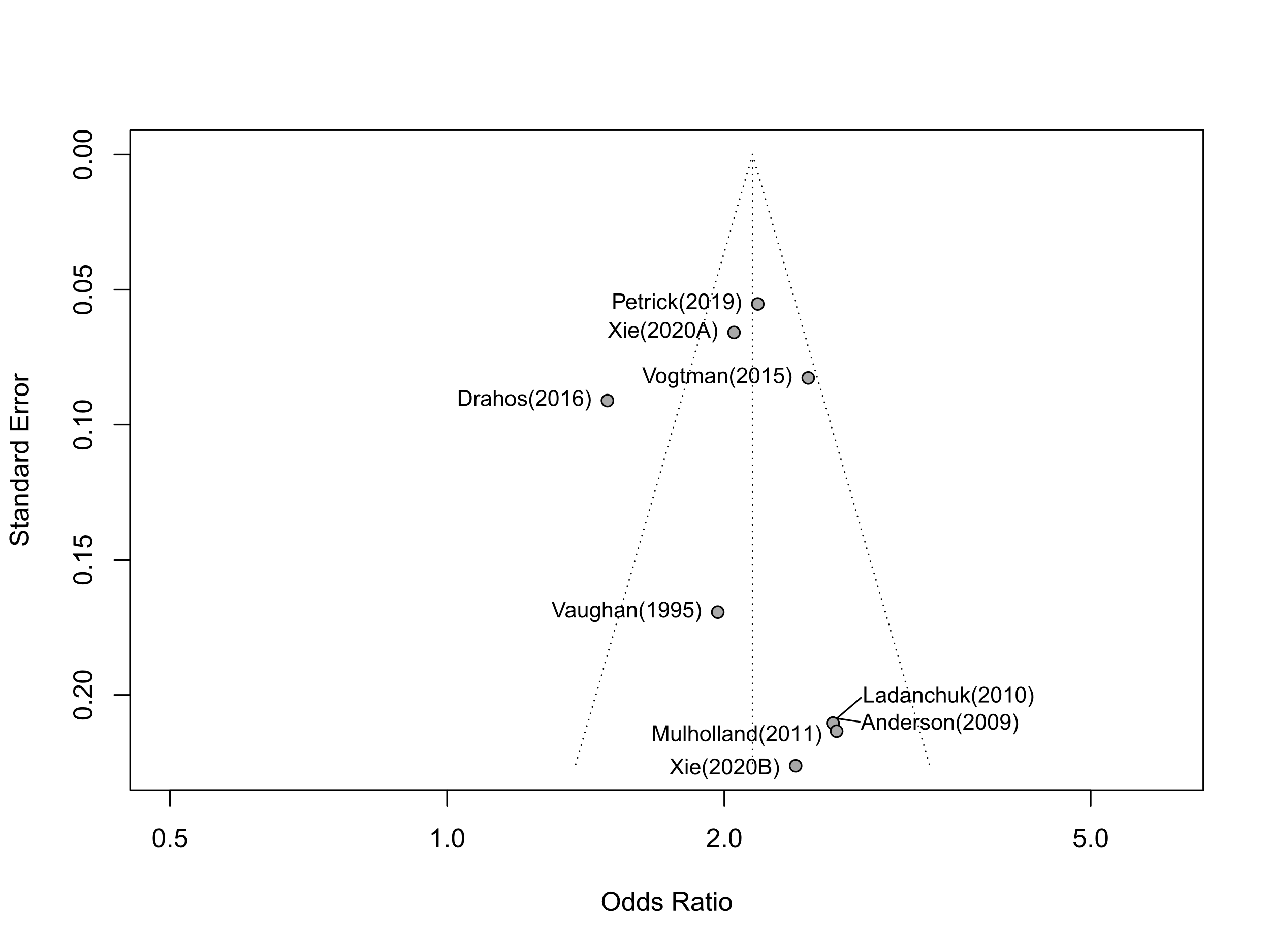


1. Funnel plot for esophageal adenocarcinoma tobacco use versus control


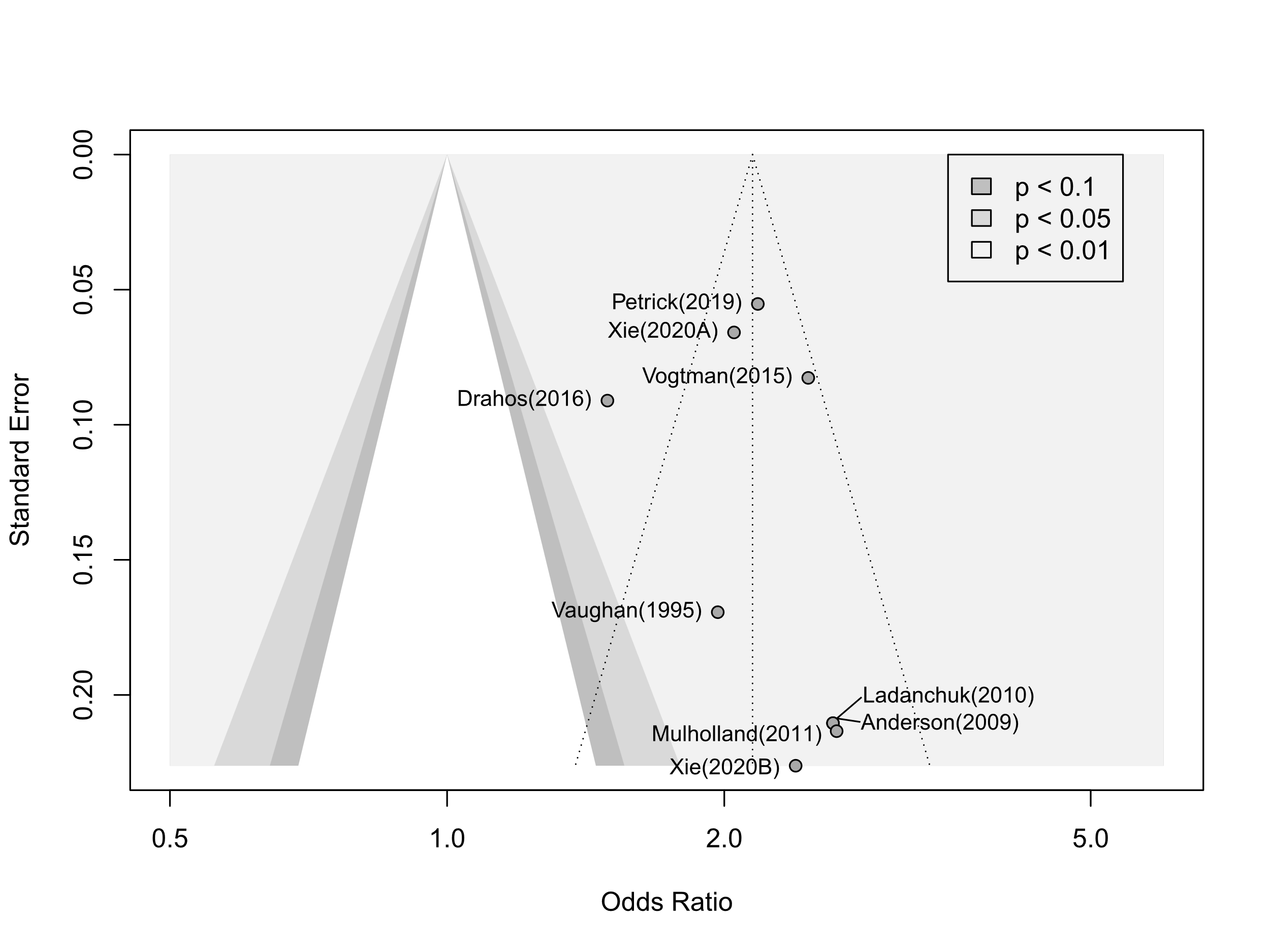


1. Contour-Enhanced funnel plot for esophageal adenocarcinoma tobacco use versus control


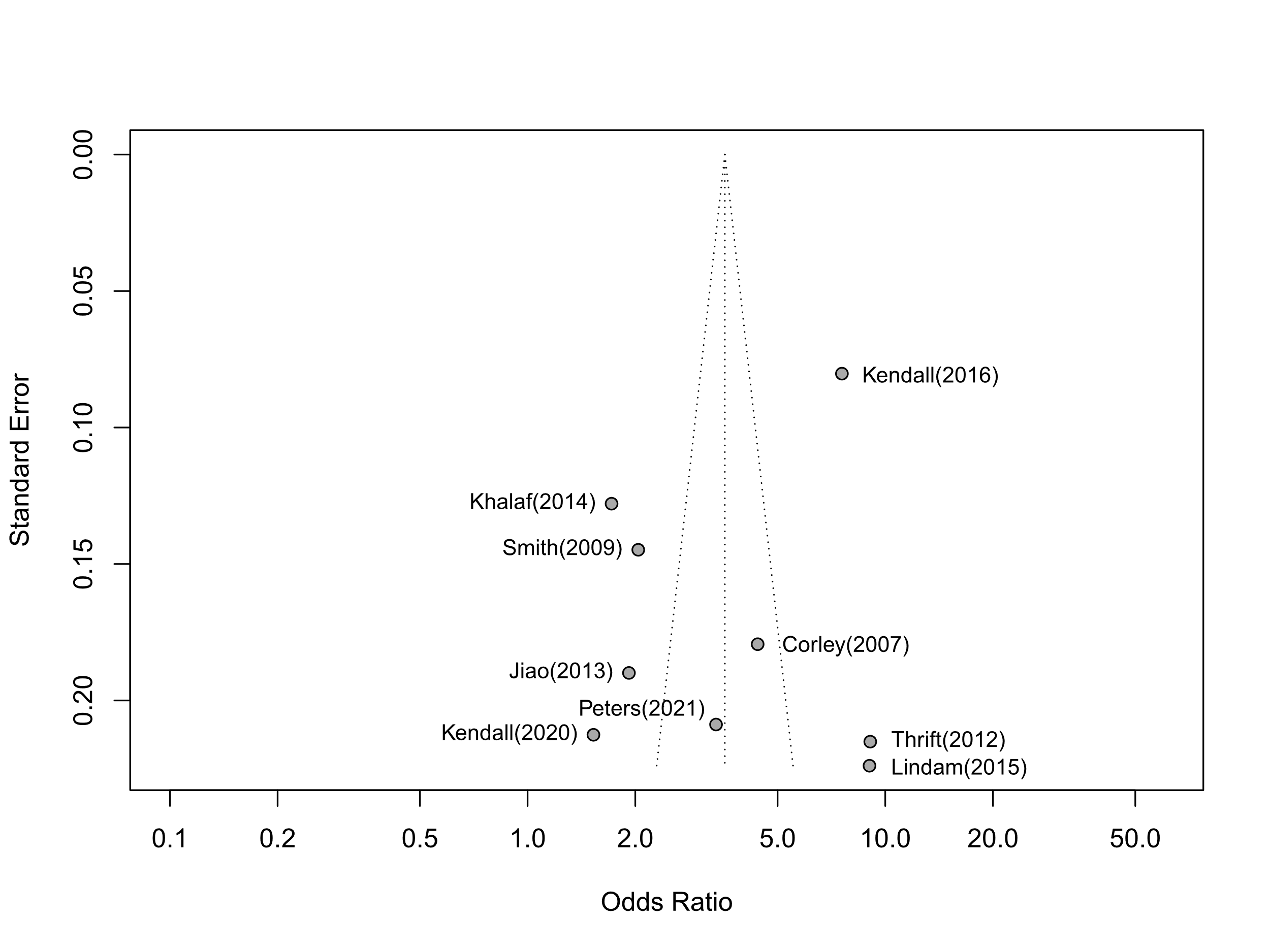


1. Funnel plot for Barrett’s esophagus weekly reflux vs control


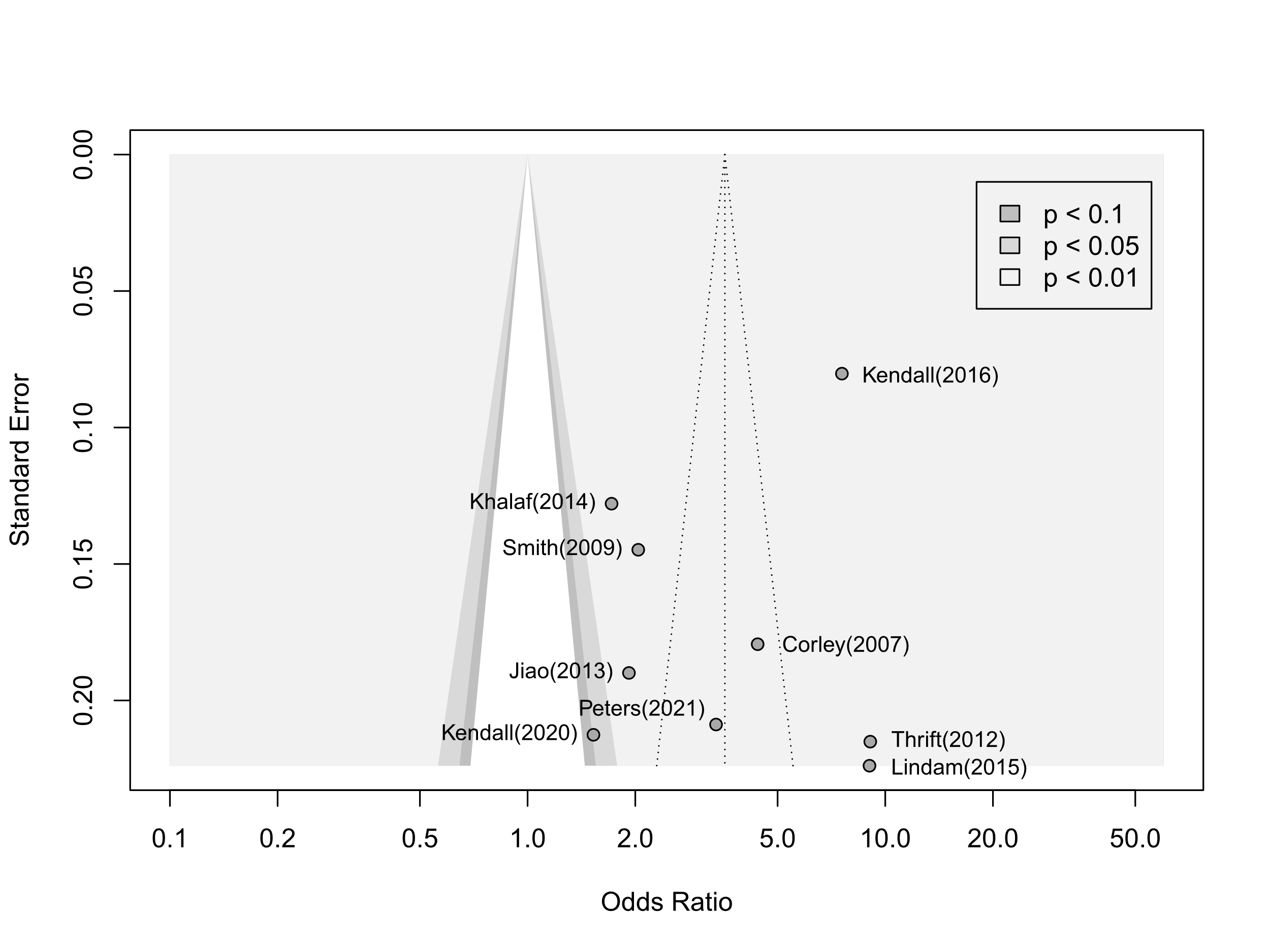


1. Contour-Enhanced funnel plot for Barrett’s esophagus weekly reflux versus control


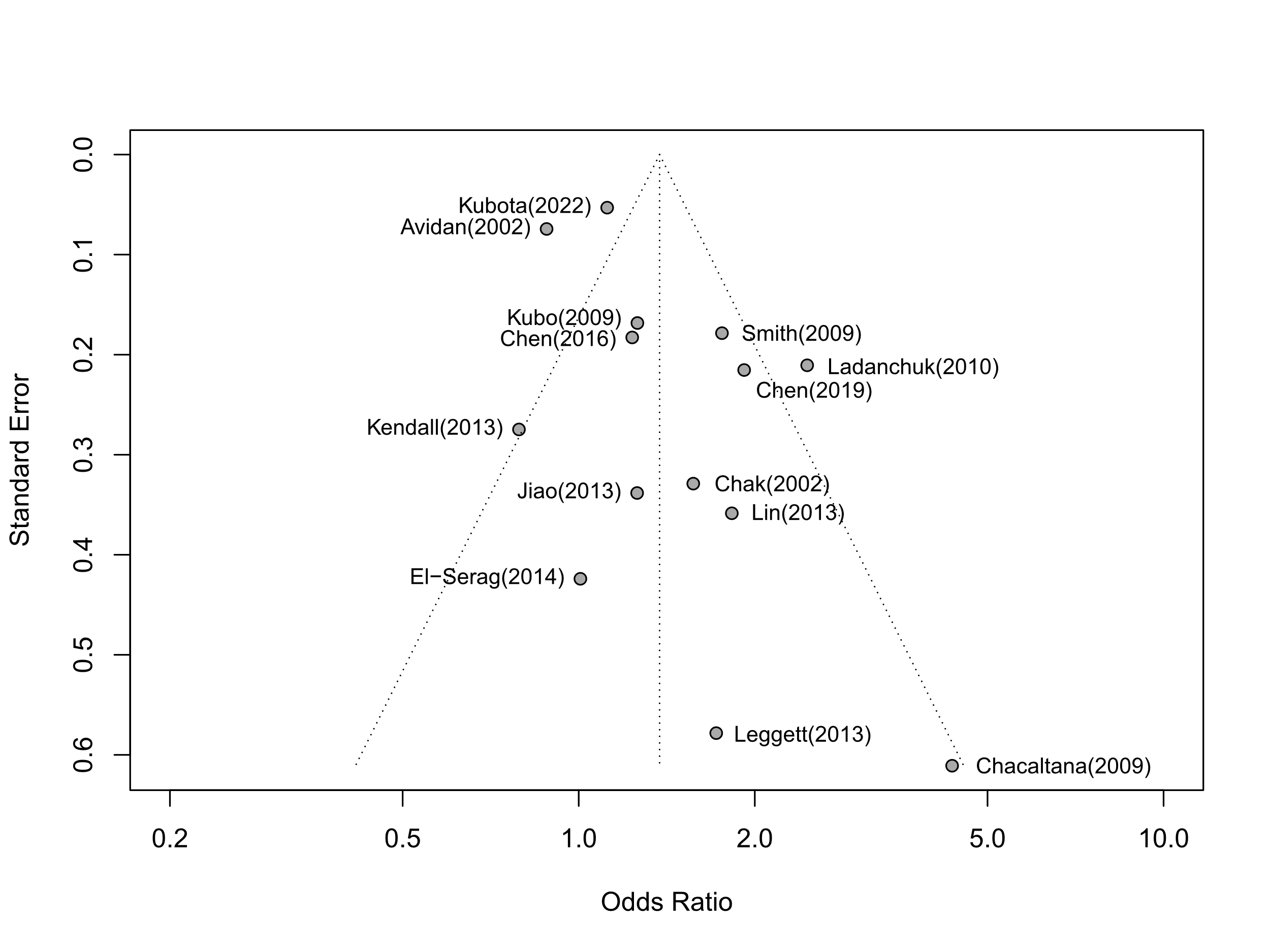


1. Funnel plot for Barrett’s esophagus alcohol use versus control


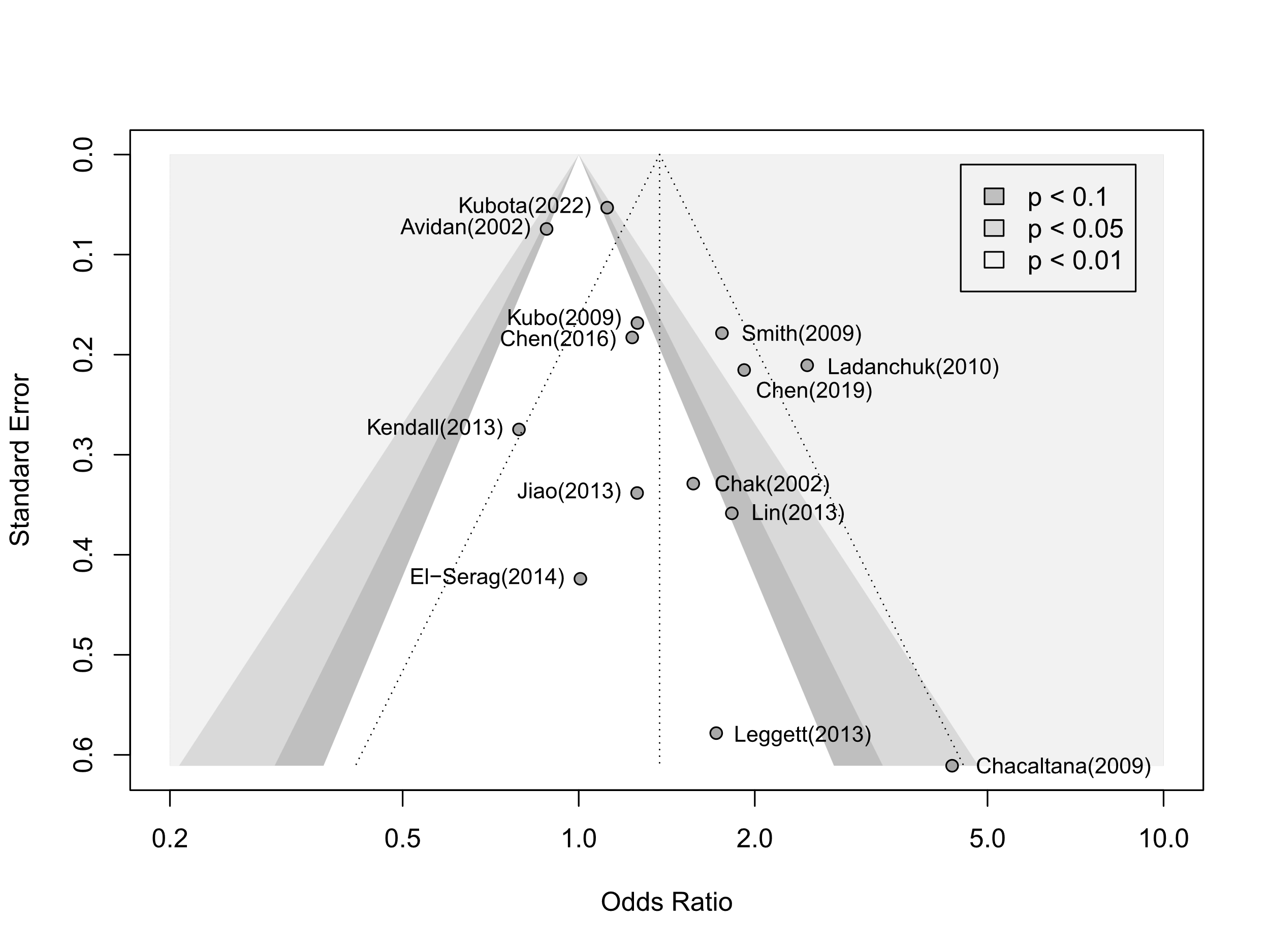


1. Contour-Enhanced funnel plot for Barrett’s esophagus alcohol use verses control


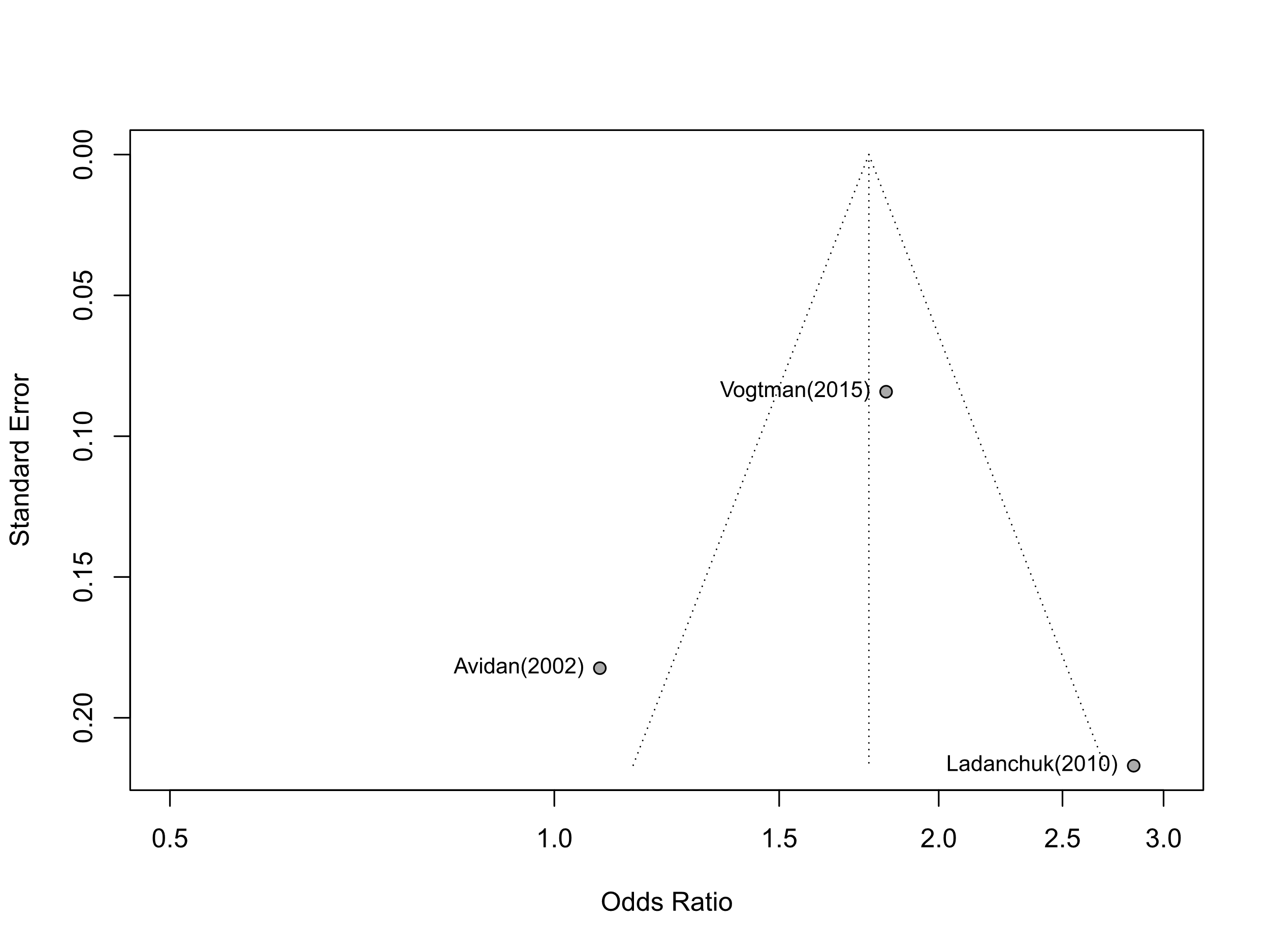


1. Funnel plot for esophageal adenocarcinoma alcohol use versus control


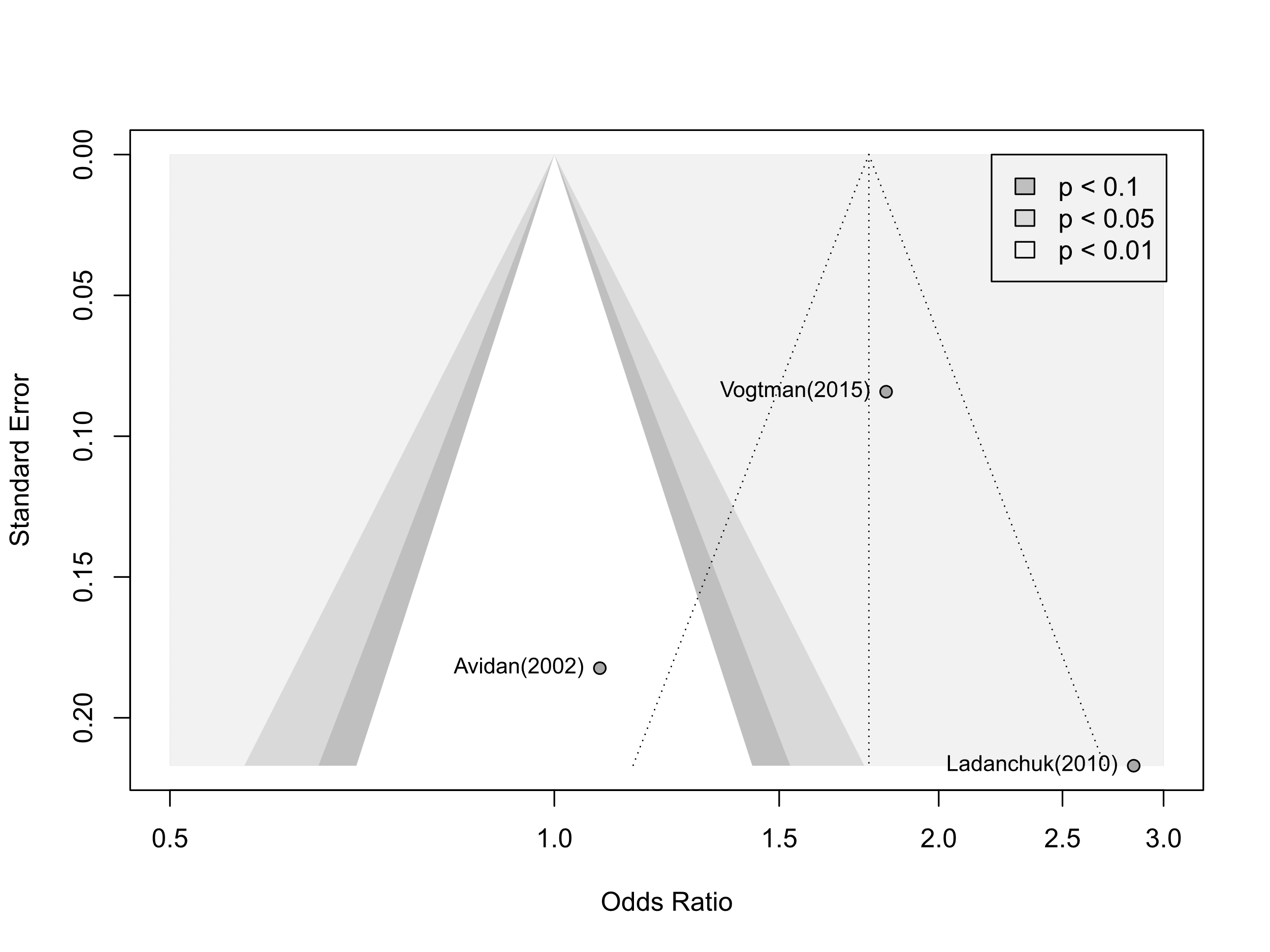


1. Contour-Enhanced funnel plot for esophageal adenocarcinoma alcohol use verses control
